# Burden of Cause-Specific Mortality Associated With PM_2.5_ Air Pollution in the United States

**DOI:** 10.1001/jamanetworkopen.2019.15834

**Published:** 2019-11-20

**Authors:** Benjamin Bowe, Yan Xie, Yan Yan, Ziyad Al-Aly

**Affiliations:** 1 Research and Education Service, Clinical Epidemiology Center, Veterans Affairs St Louis Health Care System, St Louis, Missouri; 2 College for Public Health and Social Justice, Department of Epidemiology and Biostatistics, St Louis University, St Louis, Missouri; 3 Veterans Research & Education Foundation of St Louis, St Louis, Missouri; 4 Division of Public Health Sciences, Department of Surgery, Washington University School of Medicine in St Louis, St Louis, Missouri; 5 Department of Medicine, Washington University School of Medicine in St Louis, St Louis, Missouri; 6 Nephrology Section, Medicine Service, Veterans Affairs St Louis Health Care System, St Louis, Missouri; 7 Institute for Public Health, Washington University School of Medicine in St Louis, St Louis, Missouri

## Abstract

**Importance:**

Ambient fine particulate matter (PM_2.5_) air pollution is associated with increased risk of several causes of death. However, epidemiologic evidence suggests that current knowledge does not comprehensively capture all causes of death associated with PM_2.5_ exposure.

**Objective:**

To systematically identify causes of death associated with PM_2.5_ pollution and estimate the burden of death for each cause in the United States.

**Design, Setting, and Participants:**

In a cohort study of US veterans followed up between 2006 and 2016, ensemble modeling was used to identify and characterize morphology of the association between PM_2.5_ and causes of death. Burden of death associated with PM_2.5_ exposure in the contiguous United States and for each state was then estimated by application of estimated risk functions to county-level PM_2.5_ estimates from the US Environmental Protection Agency and cause-specific death rate data from the Centers for Disease Control and Prevention.

**Main Outcomes and Measures:**

Nonlinear exposure-response functions of the association between PM_2.5_ and causes of death and burden of death associated with PM_2.5_.

**Exposures:**

Annual mean PM_2.5_ levels.

**Results:**

A cohort of 4 522 160 US veterans (4 243 462 [93.8%] male; median [interquartile range] age, 64.1 [55.7-75.5] years; 3 702 942 [82.0%] white, 667 550 [14.8%] black, and 145 593 [3.2%] other race) was followed up for a median (interquartile range) of 10.0 (6.8-10.2) years. In the contiguous United States, PM_2.5_ exposure was associated with excess burden of death due to cardiovascular disease (56 070.1 deaths [95% uncertainty interval {UI}, 51 940.2-60 318.3 deaths]), cerebrovascular disease (40 466.1 deaths [95% UI, 21 770.1-46 487.9 deaths]), chronic kidney disease (7175.2 deaths [95% UI, 5910.2-8371.9 deaths]), chronic obstructive pulmonary disease (645.7 deaths [95% UI, 300.2-2490.9 deaths]), dementia (19 851.5 deaths [95% UI, 14 420.6-31 621.4 deaths]), type 2 diabetes (501.3 deaths [95% UI, 447.5-561.1 deaths]), hypertension (30 696.9 deaths [95% UI, 27 518.1-33 881.9 deaths]), lung cancer (17 545.3 deaths [95% UI, 15 055.3-20 464.5 deaths]), and pneumonia (8854.9 deaths [95% UI, 7696.2-10 710.6 deaths]). Burden exhibited substantial geographic variation. Estimated burden of death due to nonaccidental causes was 197 905.1 deaths (95% UI, 183 463.3-213 644.9 deaths); mean age-standardized death rates (per 100 000) due to nonaccidental causes were higher among black individuals (55.2 [95% UI, 50.5-60.6]) than nonblack individuals (51.0 [95% UI, 46.4-56.1]) and higher among those living in counties with high (65.3 [95% UI, 56.2-75.4]) vs low (46.1 [95% UI, 42.3-50.4]) socioeconomic deprivation; 99.0% of the burden of death due to nonaccidental causes was associated with PM_2.5_ levels below standards set by the US Environmental Protection Agency.

**Conclusions and Relevance:**

In this study, 9 causes of death were associated with PM_2.5_ exposure. The burden of death associated with PM_2.5_ was disproportionally borne by black individuals and socioeconomically disadvantaged communities. Effort toward cleaner air might reduce the burden of PM_2.5_-associated deaths.

## Key Points

**Question:**

What are the causes of death associated with fine particulate matter (PM_2.5_) air pollution?

**Findings:**

In this cohort study of more than 4.5 million US veterans, 9 causes of death were associated with PM_2.5_ air pollution: cardiovascular disease, cerebrovascular disease, chronic kidney disease, chronic obstructive pulmonary disease, dementia, type 2 diabetes, hypertension, lung cancer, and pneumonia. The attributable burden of death associated with PM_2.5_ was disproportionally borne by black individuals and socioeconomically disadvantaged communities; 99% of the burden was associated with PM_2.5_ levels below standards set by the US Environmental Protection Agency.

**Meaning:**

This study adds to known causes of death associated with PM_2.5_ by identifying 3 new causes (death due to chronic kidney disease, hypertension, and dementia); racial and socioeconomic disparities in the burden were also evident.

## Teaser

This cohort study identifies causes of death associated with ambient fine particulate matter (PM_2.5_) air pollution in the United States and estimates the burden of death for each cause.

## Introduction

The association between ambient fine particulate matter air pollution (PM_2.5_) and risk of all-cause mortality has been well characterized.^1,2,3,4,5,6,7^ Multiple studies have outlined several specific causes of death attributable to PM_2.5_ exposure.^7^ However, a growing body of evidence (from both experimental research and human studies) suggests that the adverse health effects (including conditions associated with death) of PM_2.5_ may extend beyond those currently recognized causes of death associated with PM_2.5_ exposure.^7,8^ Evidence developed by Burnett and colleagues^8^ estimated that approximately 43% of the burden of death due to noncommunicable diseases and lower respiratory tract infections attributable to PM_2.5_ in the United States and Canada relates to causes of death that had not yet been characterized. A knowledge gap exists in that no prior study, to our knowledge, systematically examined causes of death associated with PM_2.5_ exposure, characterized their PM_2.5_ exposure-risk function, and provided estimates of their burden. In this study, we built a longitudinal cohort of 4 522 160 US veterans and studied them for 10 years; guided by evidence on the health outcomes associated with PM_2.5_, we used a systematic approach to identify causes of death associated with PM_2.5_ exposure, characterized the morphology of the association between PM_2.5_ and each cause of death, and provided estimates of the national and state-level burden of these causes.

## Methods

### Data Sources

Data on participants were obtained from United States Veterans Health Administration (VA) databases, which consist of information collected during routine care.^9,10,11,12,13,14,15,16,17^ National Death Index data contained date of death and underlying cause of death information. Modeled PM_2.5_ data for the contiguous United States were obtained from the US Environmental Protection Agency (EPA) Community Multiscale Air Quality Modeling System.^18,19^ The 2013 Area Deprivation Index (ADI), which allows for rankings of geographic locations by socioeconomic status disadvantage and is composed of education, employment, housing quality, and poverty measures, was used as a measure of a county’s socioeconomic deprivation.^20,21^ We used data from the County Health Rankings data set, a curated set of county-level determinants of health.^22^ Number of deaths due to underlying causes, defined by *International Statistical Classification of Diseases and Related Health Problems, Tenth Revision (ICD-10)* codes, were obtained from the Centers for Disease Control and Prevention WONDER online database at the state and county level in 2017.^23^ Additional information is provided in the eMethods in the Supplement. This study was reviewed and approved by the institutional review board of the VA Saint Louis Health Care System, and the requirement for informed consent was waived because risk to participants was intangible. Study reporting followed the Strengthening the Reporting of Observational Studies in Epidemiology (STROBE) reporting guideline.

### Cohort

Participants were selected if they had at least 1 record of receipt of care in the VA from January 1, 2006, to December 31, 2006, with a corresponding record of location of residence (n = 4 667 242); the last date in this time period was designated T_0_ (baseline). Participants were restricted to those who could be linked at baseline with a PM_2.5_ exposure and who had data on ADI, population density, and County Health Rankings, yielding a final cohort of 4 522 160 who were followed up until December 31, 2016.

### Exposures and Outcomes

Exposure to PM_2.5_ in 2006 was linked with a veteran’s county of residence at baseline as contained in inpatient and outpatient records of care.

Outcomes included time until death due to nonaccidental causes and noncommunicable diseases (NCDs). We further investigated associations with specific causes of death where prior literature suggested an association; for example, there is evidence that increased PM_2.5_ exposure level is associated with increased risk of chronic kidney disease, which itself is associated with increased risk of death.^10,24,25^ We analyzed death due to cardiovascular disease, cerebrovascular disease, chronic kidney disease, chronic obstructive pulmonary disease (COPD), dementia, type 2 diabetes, hypertension, lung cancer, and pneumonia.^4,10,11,26,27,28^ Cause of death was determined by the recorded *ICD-10* code for underlying cause of death. eTable 1 in the Supplement includes *ICD-10* death codes used for assignment.

### Covariates

Covariates were selected based on prior evidence of potential confounding of the association between PM_2.5_ and death.^29,30^ We adjusted for age, race, sex, smoking status, and regional characteristics of population density, ADI, percentage of population living in a rural area, percentage with limited access to healthy food, percentage with adequate access to exercise opportunities, and percentage of adults reporting excessive drinking.^31,32^ Further details are included in the eMethods in the Supplement.

### Statistical Analysis

Demographic and regional characteristics in the overall cohort and by PM_2.5_ quartile at baseline are presented as frequencies (percentage) and medians (interquartile range). Incident rates of death outcomes, standardized for age, race, sex, and smoking status, are reported for all investigated causes of death. A Kaplan-Meier curve for all-cause mortality was constructed, as well as a plot of cumulative incidence of the specific causes of death. Missing regional covariate data were imputed. Further details are included in the eMethods in the Supplement.

#### Positive and Negative Controls

Negative controls served as a means for identifying whether latent biases may be driving observed results.^33^ There is no evidence that ambient air sodium levels are associated with adverse health outcomes; here we assessed the association between ambient air sodium levels and nonaccidental causes, NCDs, cardiovascular, lung cancer, and COPD deaths (outcomes with well-established associations with PM_2.5_) using Cox proportional hazards models. We also tested a negative outcome control, accidental poisoning by exposure to noxious substance, and a positive outcome control, all-cause mortality.^3,34,35^

#### Nonlinear Exposure-Response Models

Nonlinear exposure-response models for monotonic relations were constructed.^36^ Cox proportional hazards models were estimated using linear or log-linear functions of PM_2.5_ concentration times a logistic weighting function. Multiple combinations of functions and parameters were assessed, and an optimal model (best model fit) and ensembled model are described; ensembled models were selected as primary results. Models were adjusted for all covariates. Median and 95% uncertainty intervals (UI) were obtained from 1000 bootstraps. Further information is included in the eMethods in the Supplement.

#### Sensitivity Analyses

To test robustness of study results, we built Cox models to perform the following sensitivity analyses. We (1) defined exposure by a 3-year mean of PM_2.5_ prior to baseline to broaden the time window of capturing exposure; (2) developed time-updated analyses (where exposure and outcome status were updated every quarter of a year) by defining PM_2.5_ exposure as the year prior’s mean at each point (for each time *t* during follow-up, this covers exposure from *t *− 1 year to *t*) to capture changes in PM_2.5_ over time and as participants moved from one location to another^31^ and, alternatively, building time-updated cumulative exposure analyses where we defined PM_2.5_ exposure as the cumulative mean of exposure starting from 3 years prior to baseline up to each point (for each time *t* during follow-up, this covers *t*_0_ − 3 years up to *t*)^37^; (3) varied the spatial resolution of exposure definition by assigning exposure on the basis of the nearest air monitoring within 30 and 10 miles of the participants’ residence at baseline; (4) additionally adjusted for latitude and longitude, and their interaction, as a means of accounting for geospatial correlation; and (5) additionally adjusted for ozone.^4^ Further details are provided in the eMethods in the Supplement.

#### Attributable Burden of Death Associated With PM_2.5_

Using results from the nonlinear exposure-response models, we estimated deaths associated with PM_2.5_ for each state in the contiguous United States. Owing to data availability, estimates at the county level were only done for deaths due to nonaccidental causes and NCDs. A theoretical minimum risk exposure level of 2.4 μg/m^3^ was used.^8^ For state and contiguous US burden estimates, within each state, a population-weighted risk was estimated by applying risk functions to county-level PM_2.5_ values to calculate a population-attributable fraction, which was multiplied by state-level cause-specific death values. We estimated cause-specific mortality numbers, rates per 100 000 persons, and age-standardized rates per 100 000 persons, along with 95% UIs for each value; 95% UIs were obtained from 1000 realizations of the burden. To enhance generalizability of our results, we calibrated estimates by applying an adjustment factor of the ratio of the nonaccidental cause burden estimated here to estimates calculated based on the Global Exposure Mortality Model of Burnett et al^8^ for the contiguous US.^38^ Burden was additionally estimated for deaths due to nonaccidental causes and NCD using the EPA National Ambient Air Quality Standard of 12 μg/m^3^ as the theoretical minimum risk exposure level.

#### Disparities in Burden

We estimated differences in burden by race/ethnicity category for deaths due to nonaccidental causes and NCDs. Race/ethnicity distributions were applied to the county-level estimates to estimate the attributable burden of death associated with PM_2.5_ in each race/ethnicity category. Estimates were summed across counties where data were available. Differences in burden were also estimated by ADI quartile. We analyzed the county-level age-standardized rates of death due to nonaccidental causes and NCDs associated with PM_2.5_ exposure to estimate the percentage associated with racial (percentage black or African American) and socioeconomic (ADI) disparities.^39^ We additionally conducted effect modification analyses in the nonlinear exposure-response models for deaths due to nonaccidental causes and NCDs for ADI quartile and black vs nonblack race with PM_2.5_. Results, including *P* values and the change in Akaike information criteria, are reported from the optimal model. Results were considered statistically significant at 2-tailed *P* < .05. Further information is provided in the eMethods in the Supplement. All analyses were performed in SAS Enterprise Guide statistical software version 7.1 (SAS Institute). Maps were generated using Tableau version 10.5 (Tableau Software).

## Results

There were 4 522 160 participants (4 243 462 [93.8%] male; median [interquartile range] age, 64.1 [55.7-75.5] years; 3 702 942 [82.0%] white, 667 550 [14.8%] black, and 145 593 [3.2%] other race) in the overall cohort who were followed up for a median (interquartile range) duration of 10.0 (6.8-10.2) years. The demographic characteristics of the overall cohort and by PM_2.5_ quartile are presented in Table 1. The highest quartile of PM_2.5_ exposure had the highest percentage of participants with black race, greatest proportion of current smokers, oldest median age, and greatest population density. During the course of follow-up, there were a total of 1 647 071 deaths (36.4%) (eFigure 1 in the Supplement).

**Table 1.** Demographic Characteristics of the Overall Cohort and by Baseline PM_2.5_ Quartile

Characteristic	No. (%)				
	Overall Cohort	PM_2.5_ Quartile, μg/m^3^			
		1 (4.8-10.0)	2 (10.1-11.8)	3 (11.9-13.8)	4 (13.9-20.1)
No.	4 522 160	1 167 675 (25.82)	1 122 188 (24.82)	1 134 457 (25.09)	1 097 840 (24.28)
Age, median (IQR), y	64.1 (55.7-75.5)	64.8 (56.6-75.6)	65.0 (56.5-75.7)	63.8 (55.3-75.4)	62.8 (54.0-75.0)
Male	4 243 462 (93.8)	1 097 043 (94.0)	1 054 961 (94.0)	1 064 543 (93.8)	1 026 915 (93.5)
Race					
White	3 702 942 (82.0)	1 044 988 (89.7)	971 509 (86.7)	903 470 (79.7)	782 975 (71.4)
Black	667 550 (14.8)	65 903 (5.7)	113 802 (10.2)	210 167 (18.5)	277 678 (25.3)
Other	145 593 (3.2)	54 493 (4.7)	34 993 (3.1)	20 101 (1.8)	36 006 (3.3)
Smoking status					
Current	1 130 280 (25.0)	275 293 (23.6)	266 693 (23.8)	293 062 (25.8)	295 232 (26.9)
Former	960 549 (21.2)	238 706 (20.4)	248 896 (22.2)	244 773 (21.6)	228 174 (20.8)
Never	2 431 331 (53.8)	653 676 (56.0)	606 599 (54.1)	596 622 (52.6)	574 434 (52.3)
Area Deprivation Index, median (IQR)^a^	54.7 (42.8-64.3)	54.0 (46.9-63.2)	56.1 (43.1-64.7)	57.7 (43.4-68.8)	53.6 (39.8-61.2)
Rural residence, median (IQR), %	14.5 (3.3-41.2)	20.5 (5.1-46.6)	16.8 (4.5-43.2)	23.8 (5.6-52.2)	4.6 (0.6-21.3)
Population density, median (IQR), No./square mile	284.5 (83.4-975.0)	91.8 (30.4-417.4)	247.1 (84.6-821.1)	261 (88.7-910.0)	670.0 (254.9-2344.2)
Limited access to healthy food, median (IQR), %	5.9 (3.7-8.5)	6.2 (4.3-9.6)	6.3 (4.2-8.6)	5.2 (3.2-7.9)	5.8 (2.7-7.5)
Adequate access to exercise opportunities, median (IQR), %	75.6 (57.1-90.3)	71.9 (56.3-85.7)	74.9 (53.2-87.5)	68.8 (51.9-88.4)	85.6 (69.2-95.2)
Adults reporting excessive drinking, median (IQR), %	16.5 (14.2-18.7)	16.9 (15.0-19.2)	16.9 (14.7-19.2)	14.9 (12.0-18.4)	16.5 (14.5-17.8)
Follow-up, median (IQR), y	10.0 (6.8-10.2)	10.0 (7.0-10.2)	10.0 (6.8-10.2)	10.0 (6.7-10.2)	10.0 (6.8-10.2)

Abbreviations: IQR, interquartile range; PM_2.5_, ambient fine particulate matter.

^a^ The Area Deprivation Index ranges from 0 to 100 and is a measure of socioeconomic deprivation, where higher values indicate higher levels of deprivation.

### Positive and Negative Controls

Ambient air sodium concentrations (a negative exposure control) exhibited a weak or nonsignificant association with death due to nonaccidental causes, NCDs, cardiovascular disease, COPD, and lung cancer (eTable 2 in the Supplement). Exposure to PM_2.5_ was not associated with death due to accidental poisoning by exposure to noxious substances (negative outcome control) (eFigure 2 in the Supplement). Higher levels of PM_2.5_ exposure were associated with increased risk of all-cause mortality (positive outcome control) (eFigure 2 in the Supplement).

### Causes of Death Associated With PM_2.5_ Exposure

#### Broad Causes 

**Figure 1.**
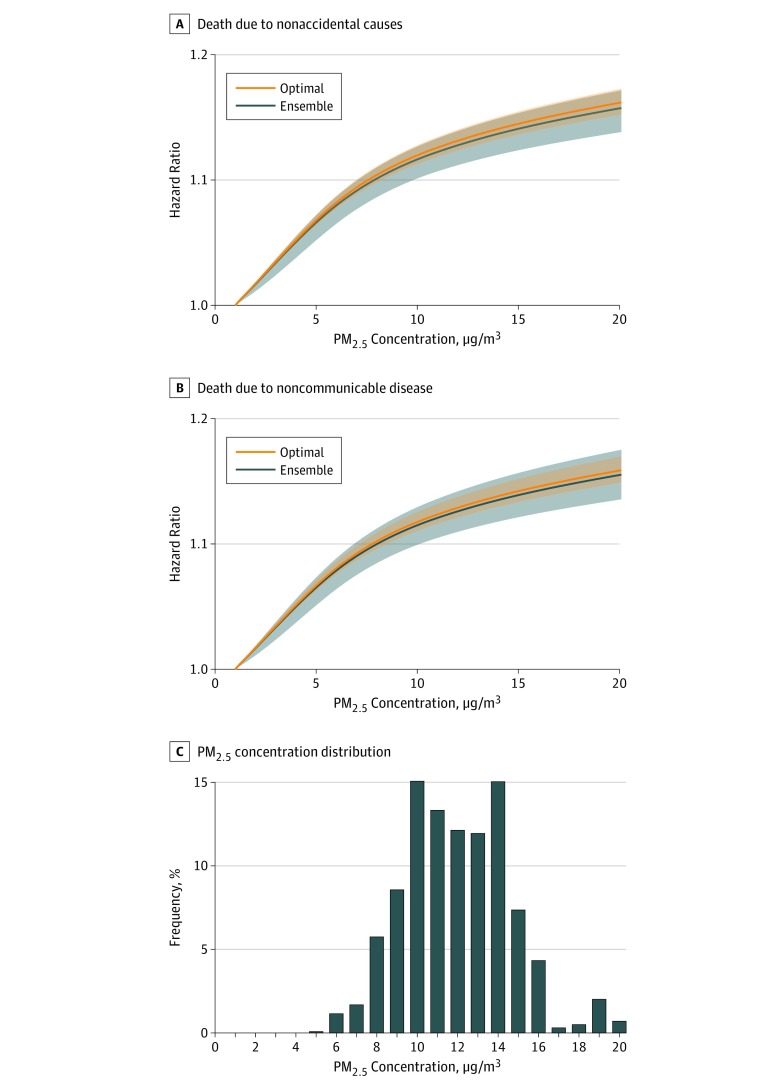
Nonlinear Exposure-Response Hazard Functions for Death Due to Nonaccidental Causes and Noncommunicable Diseases A and B, Plots are presented for both the optimal and ensembled model for nonaccidental causes (A) and noncommunicable diseases (B). The 95% uncertainty intervals are presented as bands. C, Histogram of ambient fine particulate matter (PM_2.5_) distribution.

Total number of deaths and standardized incidence rates (per 1000 person-years) in the overall cohort and by PM_2.5_ quartile are provided in eTable 3 in the Supplement. Increased PM_2.5_ concentration was associated with both risk of death due to nonaccidental causes and death due to NCDs (Figure 1). Results from the optimal model were consistent with those generated from an ensemble model for exposure-response hazard functions.

#### Specific Causes 

**Figure 2.**
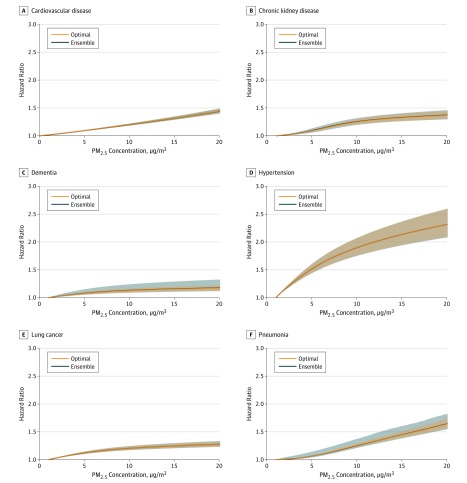
Nonlinear Exposure-Response Hazard Functions for Cause-Specific Mortality Plots are presented for both the optimal and ensembled model. The 95% uncertainty intervals are presented as bands. PM_2.5_ indicates ambient fine particulate matter.

We investigated specific causes of death due to disease states that are known to be in the causal pathway to death for which strong evidence exists of an association between PM_2.5_ exposure and the disease state.^7^ Total number of deaths and standardized incidence rates (per 1000 person-years) of these specific causes of death in the overall cohort and by PM_2.5_ quartile are provided in eTable 3 in the Supplement, and a cumulative incidence plot is furnished in eFigure 3 in the Supplement. There were associations between PM_2.5_ exposure and risk of death due to cardiovascular disease, cerebrovascular disease, chronic kidney disease, COPD, dementia, type 2 diabetes, hypertension, lung cancer, and pneumonia (6 causes are presented in Figure 2; the remaining 3, in eFigure 4 in the Supplement). Results of the optimal model were concordant with those obtained from an ensemble model for exposure-response hazard functions.

#### Sensitivity Analyses

To test robustness of study results, we conducted several sensitivity analyses (eTable 4 in the Supplement) in which we (1) used a 3-year mean PM_2.5_ exposure definition to broaden the time window to capture exposure; (2) built models with time-updated exposure (where exposure and outcome were updated every quarter year) that first accounted for changes in PM_2.5_ over time and changes in PM_2.5_ exposure levels as participants moved over the years and, alternatively, used a measure of the cumulative mean exposure to PM_2.5_ starting from 3 years before cohort entry until each point of analysis during follow-up; (3) varied the spatial resolution of exposure assignment to within 30 miles and 10 miles from the nearest air monitoring station; (4) built models additionally controlling for latitude and longitude to account for geospatial correlations; and (5) built models additionally adjusting for ozone levels. The results of the sensitivity analyses were robust to these challenges and were consistent with those in the primary analyses in that a significant association was observed between PM_2.5_ and each examined cause of death (eTable 4 in the Supplement).

### Burden of PM_2.5_-Associated Death

Uncalibrated burden estimates of death due to nonaccidental causes associated with PM_2.5_ from ensembled models for the contiguous United States were 208 500.1 deaths (95% UI, 193 285.2-225 082.6 deaths), 5.4% higher than the Global Exposure Mortality Model–based estimate. Following calibration, burden of death due to nonaccidental causes and NCDs was 197 905.1 deaths (95% UI, 183 463.3-213 644.9 deaths) and 188 540.3 deaths (95% UI, 173 883.7-209 786.3 deaths), respectively. Estimated age-standardized rates of death per 100 000 persons were 51.4 (95% UI, 47.7-55.5) and 48.4 (95% UI, 45.1-54.3) due to nonaccidental causes and NCDs, respectively (Table 2). Age-standardized death rates due to nonaccidental causes and NCDs exhibited substantial geographic variation and appeared to cluster in swaths of the Midwest, Appalachia, and the South (eFigure 5 and eFigure 6 in the Supplement).

**Table 2.** Burden of Death Associated With Ambient Fine Particulate Matter

Population	PAF, % (95% UI)	Total Deaths, No. (95% UI)	Rate, No. per 100 000 (95% UI)	Mean Age-Standardized Rate, No. per 100 000 (95% UI)
Nonaccidental cause of death				
Overall	7.76 (7.19-8.37)	197 905.1 (183 463.3-213 644.9)	61.2 (56.7-66.0)	51.4 (47.7-55.5)
Non-Hispanic black or African American	7.97 (7.41-8.60)	24 853.9 (22 728.0-27 276.1)	62.5 (57.2-68.6)	55.2 (50.5-60.6)
All other races and ethnicities				
Overall	7.73 (7.17-8.34)	172 089.7 (156 372.6-189 649.8)	60.9 (55.3-67.1)	51.0 (46.4-56.1)
Hispanic or Latino	7.89 (7.32-8.52)	30 535.7 (28 009.3-33 337.3)	53.3 (48.9-58.2)	48.9 (44.9-53.4)
White or others	7.69 (7.13-8.30)	141 553.9 (128 363.3-156 312.5)	62.8 (56.9-69.3)	51.5 (46.7-56.8)
ADI quartile^a^				
1 (2.9-53.0)	7.70 (7.13-8.31)	97 471.6 (89 406.1-106 424.4)	53.2 (48.8-58.1)	46.1 (42.3-50.4)
2 (53.1-65.5)	7.90 (7.33-8.52)	61 758.9 (56 373.5-67 823.6)	66.0 (60.3-72.5)	56.3 (51.5-61.8)
3 (65.6-75.2)	7.77 (7.21-8.38)	26 147.9 (23 359.9-29 322.2)	80.4 (71.8-90.2)	61.7 (55.1-69.2)
4 (75.3-95.9)	7.63 (7.08-8.23)	11 565.1 (9961.1-13 355.8)	84.9 (73.1-98.1)	65.3 (56.2-75.4)
Noncommunicable disease cause of death				
Overall	7.66 (7.06-8.53)	188 540.3 (173 883.7-209 786.3)	58.3 (53.7-64.8)	48.4 (45.1-54.3)
Non-Hispanic black or African American	7.88 (7.28-8.76)	23 451.7 (21 415.8- 26 769.6)	59.2 (53.9- 67.4)	52.1 (47.5-59.3)
All other races and ethnicities				
Overall	7.64 (7.04-8.50)	164 058.6 (148 247.7-187 544.2)	58.0 (52.4-66.3)	48.4 (43.8-55.3)
Hispanic or Latino	7.79 (7.19-8.68)	29 048.1 (26 502.7-32 824.8)	50.8 (46.3-57.4)	46.5 (42.4-52.5)
White or others	7.60 (7.00-8.45)	135 010.5 (121 744.9-154 719.4)	59.9 (53.9-68.6)	49.0 (44.2-56.1)
ADI quartile^a^				
1 (2.9-53.0)	7.61 (7.01-8.46)	93 066.1 (84 885.2-105 151.9)	50.8 (46.3-57.4)	43.9 (40.1-49.6)
2 (53.1-65.5)	7.80 (7.20-8.68)	58 630.1 (53 238.8-66 786.3)	62.7 (56.9-71.4)	53.3 (48.5-60.7)
3 (65.6-75.2)	7.67 (7.08-8.54)	24 907.2 (22 118.7-29 117.3)	76.6 (68.0-89.6)	58.5 (51.9-68.4)
4 (75.3-95.9)	7.54 (6.95-8.39)	10 996.9 (9420.7-13 258.3)	80.8 (69.19-97.4)	61.8 (52.9-74.6)
Cardiovascular disease	12.6 (11.7-13.5)	56 070.1 (51 940.2-60 318.3)	17.3 (16.1-18.6)	14.4 (13.3-15.5)
Cerebrovascular disease	28.4 (15.0-31.9)	40 466.1 (21 770.1-46 487.9)	12.5 (6.7-14.4)	10.6 (5.7-12.1)
Chronic kidney disease	17.1 (14.0-19.9)	7175.2 (5910.2-8371.9)	2.2 (1.8-2.6)	1.9 (1.5-2.2)
Chronic obstructive pulmonary disease	0.4 (0.2-1.6)	645.7 (300.2-2490.9)	0.2 (0.1-0.8)	0.2 (0.1-0.6)
Dementia	8.2 (6.0-13.1)	19 851.5 (14 420.6-31 621.4)	6.1 (4.5-9.8)	5.1 (3.7-8.2)
Diabetes (type 2)	1.4 (1.2-1.5)	501.3 (447.5-561.1)	0.2 (0.1-0.2)	0.1 (0.1-0.2)
Hypertension	34.1 (30.6-37.6)	30 696.9 (27 518.1-33 881.9)	9.5 (8.5-10.5)	8.0 (7.2-8.8)
Lung cancer	12.1 (10.4-14.1)	17 545.3 (15 055.3-20 464.5)	5.4 (4.7-6.3)	4.4 (3.8-5.1)
Pneumonia	18.0 (15.7-21.9)	8854.9 (7696.2-10 710.6)	2.7 (2.4-3.3)	2.3 (2.0-2.2)

Abbreviations: ADI, Area Deprivation Index; PAF, population-attributable fraction; UI, uncertainty interval.

^a^ The ADI is a measure of a county’s level of socioeconomic deprivation and ranges from 0 to 100, where 0 is low deprivation and 100 is high deprivation.

#### Burden of Death Associated With PM_2.5_ Exposure by ADI and Race

Evaluation of burden of death due to nonaccidental causes and death due to NCDs suggests that age-standardized death rates were highest among non-Hispanic black or African American individuals. Analyses by ADI quartile suggested that age-standardized death rates due to nonaccidental causes and due to NCDs increased with increasing ADI (Table 2; eFigure 7 in the Supplement).

We developed analyses to estimate the relative amount of burden associated with socioeconomic status disadvantage (expressed by ADI) and race. In models that account for both race and ADI, we estimated that in a counterfactual scenario in which racial disparities were eliminated, the age-standardized rate of death due to nonaccidental causes and death due to NCDs may be reduced by 10.6% and 10.2%, respectively; in a counterfactual scenario in which disparities related to ADI were eliminated, the age-standardized rate of death due to nonaccidental causes and death due to NCDs may be reduced by 34.5% and 34.2%, respectively.

Given the observed disparities across ADI categories and racial groups of age-standardized death rates associated with PM_2.5_, we conducted formal interaction analyses for nonlinear exposure-response models. The results suggest that the risk associated with PM_2.5_ exhibited a graded increase by increasing ADI quartile at all levels of PM_2.5_ exposure for both risk of death due to nonaccidental causes and NCDs (*P* < .001 for interaction) (eFigure 8 in the Supplement). Effect modification by race was also observed in that risk associated with PM_2.5_ increased for black individuals compared with nonblack individuals across the spectrum of PM_2.5_ exposure levels for both risk of death due to nonaccidental causes and NCDs (eFigure 8 in the Supplement).

#### Burden of Death Associated With PM_2.5_ Levels Below the Current EPA Guidelines

The EPA recommends that annual average PM_2.5_ levels not exceed 12 μg/m^3^. We estimated the burden of death associated with PM_2.5_ concentrations below the current EPA standards; the results suggest that 99.0% of the burden of death due to nonaccidental causes (195 868.0 deaths; 95% UI, 181 588.6-211 444.2 deaths) and 99.0% of the burden of death due NCDs (186 597.2 deaths; 95% UI, 172 105.3-207 614.7 deaths) were associated with PM_2.5_ levels below the current EPA guidelines (eFigure 9 in the Supplement).

#### Burden of Cause-Specific Death Associated With PM_2.5_

**Figure 3.**
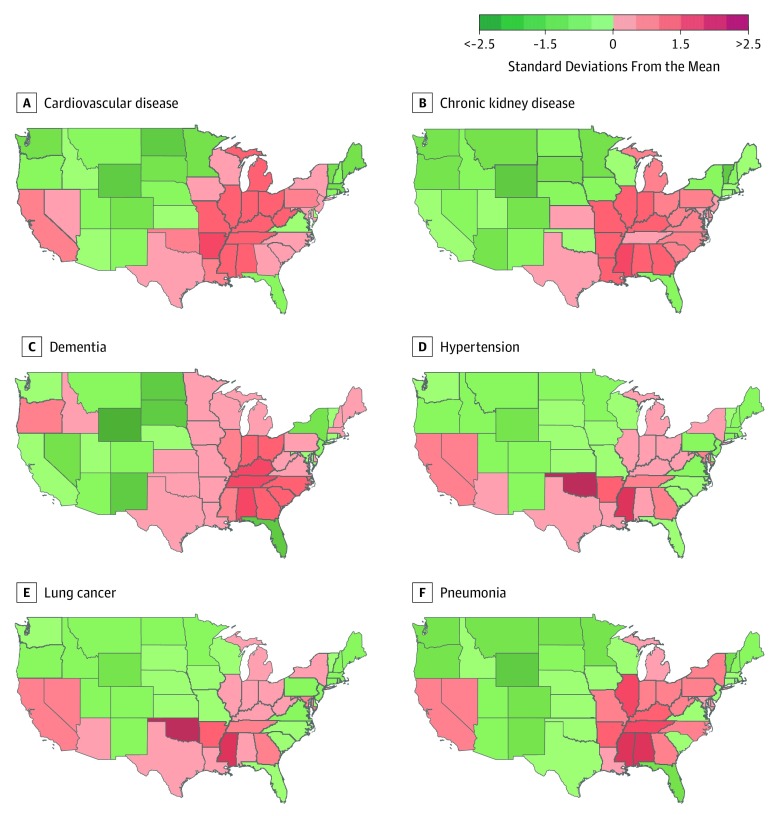
Maps of the Age-Standardized Death Rates Due to Specific Causes Associated With Ambient Fine Particulate Matter in the Contiguous United States by State Color indicates a state’s number of standard deviations from the mean for each cause of death.

Population-attributable fraction, total cause-specific death, burden of cause-specific death (per 100 000), and age-standardized burden of cause-specific death associated with PM_2.5_ are presented in Table 2. The estimated burden of cause-specific death associated with PM_2.5_ exposure was 56 070.1 deaths (95% UI, 51 940.2-60 318.3 deaths) due to cardiovascular disease, 40 466.1 deaths (95% UI, 21 770.1-46 487.9 deaths) due to cerebrovascular disease, 7175.2 deaths (95% UI, 5910.2-8371.9 deaths) due to chronic kidney disease, 645.7 deaths (95% UI, 300.2-2490.9 deaths) due to COPD, 19 851.5 deaths (95% UI, 14 420.6-31 621.4 deaths) due to dementia, 501.3 deaths (95% UI, 447.5-561.1 deaths) due to type 2 diabetes, 30 696.9 deaths (95% UI, 27 518.1-33 881.9 deaths) due to hypertension, 17 545.3 deaths (95% UI, 15 055.3-20 464.5 deaths) due to lung cancer, and 8854.9 deaths (95% UI, 7696.2-10 710.6 deaths) due to pneumonia (Table 2). Burden of cause-specific death varied by state (eTable 5 in the Supplement). Maps of 6 causes are presented in Figure 3; the remaining 3 are shown in eFigure 10 in the Supplement.

## Discussion

In this study, we systematically evaluated the association between PM_2.5_ exposure and cause-specific mortality. Exposure to PM_2.5_ has a known association with death due to cardiovascular disease, cerebrovascular disease, COPD, type 2 diabetes, lung cancer, and pneumonia. Our study expands the list of known causes of death associated with PM_2.5_ exposure to include chronic kidney disease, hypertension, and dementia. We characterized the shape of PM_2.5_ exposure-risk relationship for all causes, and the resulting estimates of cause-specific age-standardized death rates exhibited geographic variation across states in the contiguous United States. Burdens of PM_2.5_-associated death due to nonaccidental causes and NCDs were more heavily borne by non-Hispanic black and African American individuals and those living in areas with high socioeconomic deprivation; most of the burden of death due to nonaccidental causes (99%) and death due to NCDs (99%) were associated with PM_2.5_ levels below the current EPA standards.

Prior reports by the Global Burden of Disease Study^40^ and others^5^ that estimated the causes of death attributable to PM_2.5_ were limited to estimation of all-cause mortality and mortality due to ischemic heart disease, stroke, COPD, lung cancer, and lower respiratory infections; this list was most recently expanded to include diabetes. Burnett and colleagues^41^ developed an advanced Global Exposure Mortality Model that uses risk information restricted to cohort studies (41 cohorts from 16 countries) of outdoor PM_2.5_ air pollution (whereas prior work used proxy measures of PM_2.5_ exposure, including secondhand and active smoking). Their results suggested that the Global Burden of Disease study estimates vastly underestimate the burden of all-cause and cause-specific mortality, and that PM_2.5_ exposure may be related to additional causes of death other than those currently considered by the Global Burden of Disease study.^8^ In our study, we leveraged the enhanced understanding provided by Burnett et al^41^ and systematically evaluated specific causes of death where there is evidence of an association between PM_2.5_ and the underlying disease state. Our findings identified additional causes including death due to chronic kidney disease, dementia, and hypertension and provide updated estimates for all 9 causes for the contiguous United States. Evidence from Burnett et al^41^ suggests a 43% gap between the estimated burden of all-cause mortality and burden estimates of currently recognized specific causes of death associated with PM_2.5_ exposure; this gap has since been narrowed with the recent inclusion of diabetes.^40,41^ The work presented here suggests that the recognition of 3 additional causes of death associated with PM_2.5_ exposure further shrinks this gap to 8%, representing an overall improvement but also suggesting that a smaller gap remains a likely indication that burden of some causes may be underestimated or that there are yet-to-be identified causes that are not accounted for in our analyses.

Evidence from this work suggests that burden of death associated with PM_2.5_ exposure concentrates geographically in the Midwest, Appalachia, and the South and is disproportionally borne by non-Hispanic black and African American individuals and those living in counties with a high index of socioeconomic deprivation. Our analyses of counterfactual scenarios suggest that both race and ADI contribute measurably and independently to burden of death associated with PM_2.5_ exposure. The findings suggest that the underlying socioeconomic conditions (independent of race) in which people live and disparities based on race (independent of ADI) are both important factors in the burden of death associated with PM_2.5_. Disparities in PM_2.5_-associated age-standardized death rates reflect the influence of not only differences in PM_2.5_ exposure and underlying mortality rates, but also sensitivity to exposure. Profound racial and socioeconomic disparities in PM_2.5_ exposure are well documented; our formal interaction analyses provide evidence suggesting that for the same level of PM_2.5_ exposure, black individuals and those living in disadvantaged communities (areas of high ADI) are more vulnerable (exhibit higher risk) to the adverse health outcomes associated with PM_2.5_ exposure,^3,42^ further compounding their risk. Greater attention is needed to address and alleviate the burden borne by racial monitories and those living in disadvantaged communities who might also be least equipped to deal with the adverse health consequences of air pollution.^43,44,45,46^

There is a considerable national discussion about the current EPA standards for air pollutants and whether further reduction might yield improved health outcomes.^3,47,48^ An extensive body of scientific evidence suggests substantial health gains realized by cleaner air, and that further reduction in PM_2.5_ might lead to even greater reduction in burden of disease.^49^ Our results further inform this national discussion in that the shape of the exposure-risk function for most causes of death suggests increased risk across the full PM_2.5_ range between the theoretical minimum risk exposure level and 12 μg/m^3^ (the current EPA standard). We estimated the number of deaths associated with PM_2.5_ for the entire spectrum of exposure levels experienced by people living in the United States. Our analyses suggest that substantial burden of death due to nonaccidental causes (99%) and death due to NCDs (99%) are associated with PM_2.5_ levels below the current EPA standard of 12 μg/m^3^ (eFigure 9 in the Supplement). This result reflects a near total elimination of death burden associated with PM_2.5_ concentrations above 12 μg/m^3^, a testament to the remarkable progress in cleaning the air and meeting the current EPA standards, but also indicates that further reduction in PM_2.5_ concentrations below the current EPA standards may yield additional public health benefit.

### Limitations and Strengths

This study has several limitations. We present burden estimates derived from a cohort of US veterans in which the majority of participants were older white men, which may limit generalizability of study results; although we used estimates from a state-of-the-art multistudy integrative metaregression to calibrate our nonaccidental burden estimate, estimates of other causes (which applied the same calibration factor) may have had different proportions of error. Although we accounted for several individual-level and county-level health characteristics, our analyses do not account for individual-level differences in socioeconomic status, physical activity, and indoor exposure to air pollution; however, the successful application of negative exposure controls, a negative outcome control, and a positive outcome control lessens the concern about residual confounding. Underlying cause of death codes from the National Death Index may contain some misclassification,^50^ and our analytic approach did not consider multiple causes of death simultaneously; however, our estimates of death due to nonaccidental causes were calibrated against those of Burnett and colleagues.^8^ Our analyses did not consider the source or the chemical composition and toxic content of PM_2.5_, which might vary geographically; however, studies have shown that estimates using nonspecific PM_2.5_ biomass alone will underestimate the burden of disease attributable to PM_2.5_ pollution.^5^ Although we developed strategies to account for cumulative exposure (averaging exposure values starting from 3 years prior to cohort up to each point of analysis during follow-up), our data did not account for complete lifetime history of exposure. Our study focused on evaluating causes of death associated with PM_2.5_ exposure; however, evaluation of causes of death associated with exposure to other pollutants should be undertaken in future research.

Our study also has several strengths. Guided by evidence in the literature on health effects of PM_2.5_, we systematically evaluated the morphology of the relationship between PM_2.5_ and specific causes of death in a national cohort of more than 4.5 million people followed for a median duration of 10 years, which provides power to detect associations that may not be feasible in smaller cohorts. We also developed and tested negative exposure, negative outcome, and positive outcome controls to investigate concerns about spurious associations. We used state-of-the-art methods to estimate health burden and provided estimates of burden at the county level for deaths due to nonaccidental causes and NCDs and state level for specific causes of death. We provided estimates of uncertainty that incorporate not only the standard error of parameter estimates, but uncertainty due to model construction and standard error in National Death Index death rate estimates.^29,30^

## Conclusions

In conclusion, we provide evidence of an association between PM_2.5_ air pollution and 9 causes of death—expanding by 3 the list of specific causes of death associated with ambient particulate matter air pollution. We characterize the shape of the association and provide measures of burden for each specific cause at the national and state level. Our results provide further evidence that racial disparities and nonracial socioeconomic disparities contribute measurably and independently to the burden of death associated with PM_2.5_ exposure. Finally, we provide estimates that nearly all deaths attributable to air pollution in the contiguous United States are associated with ambient air pollution concentrations below the current EPA standards, a finding that both reflects past success and suggests that more stringent PM_2.5_ air quality standards may further reduce the national death toll associated with air pollution.

## Notes

Supplement. **eMethods.** Additional Information on Methods **eReferences** **eTable 1.** ICD-10 Cause of Death Codes Used for Cause Categories and Specific Causes **eTable 2.** Association of Ambient Fine Sodium With Cause-Specific Mortality (N=2,167,831) **eTable 3.** Total Events and Incidence Rates of Deaths (per 1,000 Person-Years) Overall and by Baseline PM_2.5_ Quartile Standardized for Age, Race, Sex, and Smoking Status **eTable 4.** Sensitivity Analyses of the Association Between PM_2.5_ and Cause-Specific Mortality **eTable 5.** Cause Specific Death Associated With PM_2.5_ in the Contiguous US and by State **eFigure 1.** Kaplan-Meier Curve of All-Cause Mortality **eFigure 2.** Non-linear Exposure-Response Hazard Functions for Negative and Positive Outcome Controls **eFigure 3.** Cumulative Incidence of Specific Causes of Death **eFigure 4.** Non-linear Exposure-Response Hazard Functions for Cause-Specific Mortality **eFigure 5.** Map of the Age-Standardized Death Rates Due to Non-accidental Causes Associated With PM_2.5_ in the Contiguous United States by County **eFigure 6.** Map of the Age-Standardized Death Rates Due to Non-communicable Diseases Associated With PM_2.5_ in the Contiguous United States by County **eFigure 7.** Age-Standardized Death Rates Due to Non-accidental Causes and Non-communicable Diseases Associated With PM_2.5_ by County Area Deprivation Index and Percentage Black or African American **eFigure 8.** Effect Modification of the Association of PM_2.5_ With Risk of Death Due to Non-accidental Causes and Non-communicable Diseases by ADI Quartile and Black Race **eFigure 9.** Burden of Death Due to Non-accidental Causes and Non-communicable Diseases Associated With PM_2.5_ in the Contiguous US **eFigure 10.** Maps of the Age-Standardized Death Rates Due to Specific Causes Associated With PM_2.5_ in the Contiguous United States by State
