# Supplementary material for: Burden of Cause-Specific Mortality Associated With PM2.5 Air Pollution in the United States
Source: JAMA Netw Open. 2019 Nov 20;2(11):e1915834. doi: 10.1001/jamanetworkopen.2019.15834 (PMC6902821; doi:10.1001/jamanetworkopen.2019.15834)
Supplement: Supplement. — eMethods. Additional Information on Methods eReferences eTable 1. ICD-10 Cause of Death Codes Used for Cause Categories and Specific Causes eTable 2. Association of Ambient Fine Sodium With Cause-Specific Mortality (N=2,167,831) eTable 3. Total Events and Incidence Rates of Deaths (per 1,000 Person-Years) Overall and by Baseline PM2.5 Quartile Standardized for Age, Race, Sex, and Smoking Status eTable 4. Sensitivity Analyses of the Association Between PM2.5 and Cause-Specific Mortality eTable 5. Cause Specific Death Associated With PM2.5 in the Contiguous US and by State eFigure 1. Kaplan-Meier Curve of All-Cause Mortality eFigure 2. Non-linear Exposure-Response Hazard Functions for Negative and Positive Outcome Controls eFigure 3. Cumulative Incidence of Specific Causes of Death eFigure 4. Non-linear Exposure-Response Hazard Functions for Cause-Specific Mortality eFigure 5. Map of the Age-Standardized Death Rates Due to Non-accidental Causes Associated With PM2.5 in the Contiguous United States by County eFigure 6. Map of the Age-Standardized Death Rates Due to Non-communicable Diseases Associated With PM2.5 in the Contiguous United States by County eFigure 7. Age-Standardized Death Rates Due to Non-accidental Causes and Non-communicable Diseases Associated With PM2.5 by County Area Deprivation Index and Percentage Black or African American eFigure 8. Effect Modification of the Association of PM2.5 With Risk of Death Due to Non-accidental Causes and Non-communicable Diseases by ADI Quartile and Black Race eFigure 9. Burden of Death Due to Non-accidental Causes and Non-communicable Diseases Associated With PM2.5 in the Contiguous US eFigure 10. Maps of the Age-Standardized Death Rates Due to Specific Causes Associated With PM2.5 in the Contiguous United States by State [file jamanetwopen-2-e1915834-s001.pdf]

Supplementary Online Content

Bowe B, Xie Y, Yan Y, Al-Aly Z. Burden of cause-specific mortality associated with $PM_{2.5}$ air pollution in the United States. *JAMA Netw Open*. 2019;2(11):e1915834. doi:10.1001/jamanetworkopen.2019.15834

eMethods. Additional Information on Methods

eReferences

eTable 1. ICD-10 Cause of Death Codes Used for Cause Categories and Specific Causes

eTable 2. Association of Ambient Fine Sodium With Cause-Specific Mortality (N=2,167,831)

eTable 3. Total Events and Incidence Rates of Deaths (per 1,000 Person-Years) Overall and by Baseline $PM_{2.5}$ Quartile Standardized for Age, Race, Sex, and Smoking Status

eTable 4. Sensitivity Analyses of the Association Between $PM_{2.5}$ and Cause-Specific Mortality

eTable 5. Cause Specific Death Associated With $PM_{2.5}$ in the Contiguous US and by State

eFigure 1. Kaplan-Meier Curve of All-Cause Mortality

eFigure 2. Non-linear Exposure-Response Hazard Functions for Negative and Positive Outcome Controls

eFigure 3. Cumulative Incidence of Specific Causes of Death

eFigure 4. Non-linear Exposure-Response Hazard Functions for Cause-Specific Mortality

eFigure 5. Map of the Age-Standardized Death Rates Due to Non-accidental Causes Associated With $PM_{2.5}$ in the Contiguous United States by County

eFigure 6. Map of the Age-Standardized Death Rates Due to Non-communicable Diseases Associated With $PM_{2.5}$ in the Contiguous United States by County

eFigure 7. Age-Standardized Death Rates Due to Non-accidental Causes and Non-communicable Diseases Associated With $PM_{2.5}$ by County Area Deprivation Index and Percentage Black or African American

eFigure 8. Effect Modification of the Association of $PM_{2.5}$ With Risk of Death Due to Non-accidental Causes and Non-communicable Diseases by ADI Quartile and Black Race

eFigure 9. Burden of Death Due to Non-accidental Causes and Non-communicable Diseases Associated With $PM_{2.5}$ in the Contiguous US

eFigure 10. Maps of the Age-Standardized Death Rates Due to Specific Causes Associated With $PM_{2.5}$ in the Contiguous United States by State

© 2019 Bowe B et al. *JAMA Network Open.*

This supplementary material has been provided by the authors to give readers additional information about their work.

© 2019 Bowe B et al. *JAMA Network Open.*

eMethods: Additional Information on Methods

Data Sources

Demographics and location of residence at time of receipt of care was collected from the MedSAS datasets. Smoking status was obtained from the CDW Health Factors domain. Demographics were additionally obtained from the Vital Status file. Data on date of death and underlying cause of death was obtained from the VA Suicide Registry, which links VA records with the National Death Index. Population density at the county level was obtained from the US Census Bureaus' Small Area Income and Poverty Estimates (SAIPE) data. Distribution of race and ethnicity in 2017 for each county was obtained from the 2017 American Community Survey 5-years estimates[1].

Covariates

Smoking status was categorized as never, former, or current smoker[2]. Race was categorized as white, black, or other. The Area Deprivation Index was summarized at the county level.

Statistical Analyses

Incidence rates were standardized to the distribution of age, race, sex, and smoking status in the overall cohort. Stochastic imputation based on the fully conditional specification method was used to impute missing regional covariates using all domains of the CHR, ADI, population density, splines of latitude and longitude, and a state indicator[3].

Non-linear exposure-response models

© 2019 Bowe B et al. *JAMA Network Open.*

Prior literature has suggested that the relation between $PM_{2.5}$ and health outcomes can follow a non-linear form[4]. However traditional non-linear analytic methods, such as splines, may result in non-monotonic relations that are biologically implausible[5]. In this study we applied the analytic framework put forth by Nasari and colleagues[5]. Briefly, Cox proportional hazard models with non-linear exposure-responses are constructed. Multiple models that allow for a wide variety of monotonic relations between $PM_{2.5}$ and death were explored.

The analyses are done using Cox proportional hazard models of the form:

$$\lambda(t|x,z) = \lambda_0(t) * \exp(\gamma' x + \beta * w(z|\mu,\tau) * f(z))$$

where $\lambda_0(t)$ is the baseline hazard at time t, and γ' the vector of coefficients for covariate vector x. The term $\beta * w(z|\mu,\tau) * f(z)$ relates the $PM_{2.5}$ (z) to the hazard of the outcome. β is the coefficient, $w(z|\mu,\tau)$ a logistic weighting function, and $f(z)$ a function of $PM_{2.5}$. The logistic weighting function is:

$$w(z|\mu,\tau) = \{1 + \exp(-\left(\frac{z-\mu}{\tau * r}\right))\}^{-1}$$

where μ is a location parameter, τ a parameter that controls the curvature of the weighting function, and r the range of *z.* The function $f(z)$ may be either the identity or a natural log transformation of *z*. The analysis tests multiple iterations of the model, where μ, τ, and $f(z)$ are varied. Model fit, based on the – 2LL is used to guide model construction until optimal fit is achieved. Beyond the optimal model, the top three best performing models are jointly used to construct an ensembled estimate, where models are weighted by differences in the likelihood. Data is trimmed at 1% tails during estimation to enhance model stability. Additional details are provided elsewhere, as is a SAS macro for conducting analyses using this method[5]. Continuous covariates were treated as natural cubic splines.

© 2019 Bowe B et al. *JAMA Network Open.*

Sensitivity Analyses

Time updated analyses we done through December 31, 2014, due to $PM_{2.5}$ estimate availability. A time period of a quarter of a year was used. Latitude and longitude were assigned on the basis of the latitude and longitude of the centroid of the participants county of residence. The Haversine equation was used in to calculated distance to the nearest air monitoring station. Ozone values were assigned on the basis of the nearest air monitoring station measuring ozone levels within 30 miles of the participants residence of location.

Attributable burden of death

Data values used in the estimation of burden were 2014 EPA $PM_{2.5}$ values, the most recent estimates that were available, and 2017 CDC underlying death values, the most recent data available at the time of manuscript preparation.

The GEMM based estimate for the contiguous US was derived by combining age specific non-communicable and lower respiratory infection death parameters estimates with 2014 EPA $PM_{2.5}$ data and 2017 CDC death numbers by age range for each state, then summed[6,7].

Disparities in burden

Disparities in burden were estimated by linking county level burden estimates with 2017 ACS 5-year population estimates. The percent in a county of each race/ethnicity were applied to the estimated burden of death associated with $PM_{2.5}$, and then combined to get overall burden in that race/ethnicity. For rates, population weighted averages were computed. This approach allows for quantification of the burden at the county level, leveraging the resolution of our estimates, however it assumes that death rates are equal amongst all race/ethnicities in the same county; this approach was taken due to limited data by race at the county level for death rates. ADI quartile was defined at the county level.

© 2019 Bowe B et al. *JAMA Network Open.*

A negative binomial regression model with a log link was built, and the incident rate ratios were used in estimating the percentage associated using a PAF equation using 0 as the counterfactual scenario values for ADI and percent black or African American[8]. In this instance this is meant to represent a scenario where there are no disparities (that is where there are no differences in burden associated with differences in a county's racial or socio-economic makeup). An interaction between ADI and percentage black or African American suggested some overlap in effect (negative beta, $p<0.001$); we opted to incorporate the interaction into the racial disparity burden, as evidence suggests that a portion of observed racial disparities is attributable to socioeconomic disparities. Interactions with a p-value <0.05, and improved Akaike Information Criteria (AIC) were taken as evidence of effect modification. A negative change in AIC indicates an improvement in model fit.

eReferences

1. 2013-2017 American Community Survey 5-Year Estimates.
2. McGinnis KA, Brandt CA, Skanderson M, et al. Validating smoking data from the Veteran's Affairs Health Factors dataset, an electronic data source. *Nicotine & Tobacco Research.* 2011;13(12):1233-1239.
3. Van Buuren S. Multiple imputation of discrete and continuous data by fully conditional specification. *Statistical methods in medical research.* 2007;16(3):219-242.
4. Cohen AJ, Brauer M, Burnett R, et al. Estimates and 25-year trends of the global burden of disease attributable to ambient air pollution: an analysis of data from the Global Burden of Diseases Study 2015. *The Lancet.* 2017;389(10082):1907-1918.
5. Nasari MM, Szyszkowicz M, Chen H, et al. A class of non-linear exposure-response models suitable for health impact assessment applicable to large cohort studies of ambient air pollution. *Air Quality, Atmosphere & Health.* 2016;9(8):961-972.
6. Lelieveld J, Klingmüller K, Pozzer A, et al. Cardiovascular disease burden from ambient air pollution in Europe reassessed using novel hazard ratio functions. *European heart journal.* 2019.
7. Burnett R, Chen H, Szyszkowicz M, et al. Global estimates of mortality associated with long-term exposure to outdoor fine particulate matter. *Proceedings of the National Academy of Sciences.* 2018;115(38):9592-9597.
8. Jaakkola MS, Jaakkola JJ. Assessment of public health impact of work-related asthma. *BMC medical research methodology.* 2012;12(1):22.

© 2019 Bowe B et al. *JAMA Network Open.*

eTable 1: ICD-10 cause of death codes used for cause categories and specific causes

Cause	ICD10 Codes
Non-accidental	A00-R99
Non-communicable diseases	C00-N99, R00-R99
Cerebrovascular disease	I60-I69
Chronic kidney disease	N18-N19
Chronic obstructive pulmonary disease	J40-J44
Dementia	F01-F03, G30, R54
Diabetes (Type 2)	E11
Hypertension	I10-I15
Cardiovascular disease	I20-I25, I50
Lung cancer	C33-C34
Pneumonia	J12-J18

eTable 2: Association of ambient fine sodium with cause-specific mortality (N=2,167,831)

Cause	Hazard Ratio[a] (95% CI)
Non-accidental causes	1.01 (1.00-1.01)
Non-communicable diseases	1.01 (1.00-1.01)
Cardiovascular disease	1.02 (1.02-1.03)
Chronic obstructive pulmonary disease	1.00 (0.99-1.01)
Lung cancer	1.00 (0.99-1.01)

[a]For every one IQR (0.072 μg/m^3) increase
CI, confidence interval

© 2019 Bowe B et al. *JAMA Network Open.*

eTable 3: Total events and incidence rates of deaths (per 1,000 person-years) overall and by baseline $PM_{2.5}$ quartile standardized for age, race, sex, and smoking status

**Cause**	**Total events (%)**	**Incidence rate (95% CI)**			
		**$PM_{2.5}$ quartile 1: 4.8-10.0 (μg/m³)**	**$PM_{2.5}$ quartile 2: 10.1-11.8 (μg/m³)**	**$PM_{2.5}$ quartile 3: 11.9-13.8 (μg/m³)**	**$PM_{2.5}$ quartile 4: 13.9-20.1 (μg/m³)**
Non-accidental	1,570,798 (34.74)	25.48 (25.31-25.66)	26.09 (25.90-26.27)	26.78 (26.60-26.97)	27.03 (26.85-27.22)
Non-communicable diseases	1,526,798 (33.76)	24.49 (24.32-24.66)	25.00 (24.82-25.18)	25.63 (25.45-25.82)	25.87 (25.69-26.05)
Cardiovascular disease	340,365 (7.53)	3.97 (3.90-4.03)	4.21 (4.14-4.28)	4.37 (4.30-4.44)	4.48 (4.40-4.55)
Cerebrovascular disease	71,771 (1.6)	1.12 (1.08-1.15)	1.14 (1.11-1.18)	1.17 (1.14-1.21)	1.19 (1.15-1.23)
Chronic Kidney Disease	29,016 (0.64)	0.36 (0.34-0.38)	0.42 (0.39-0.44)	0.48 (0.45-0.51)	0.44 (0.42-0.47)
Chronic obstructive pulmonary disease	124,294 (2.8)	1.65 (1.61-1.70)	1.57 (1.53-1.61)	1.54 (1.50-1.58)	1.53 (1.49-1.57)
Dementia	103,286 (2.3)	0.68 (0.66-0.70)	0.69 (0.67-0.71)	0.68 (0.66-0.70)	0.70 (0.68-0.72)
Diabetes (Type 2)	14,448 (0.3)	0.29 (0.26-0.31)	0.25 (0.24-0.27)	0.24 (0.22-0.26)	0.27 (0.25-0.29)
Hypertension	37,169 (0.8)	0.84 (0.81-0.87)	0.88 (0.84-0.91)	0.85 (0.82-0.89)	0.99 (0.96-1.03)
Lung cancer	128,469 (2.8)	1.89 (1.84-1.94)	2.03 (1.98-2.09)	2.12 (2.07-2.18)	2.02 (1.97-2.07)
Pneumonia	33,099 (0.7)	0.33 (0.31-0.35)	0.38 (0.36-0.40)	0.43 (0.41-0.45)	0.46 (0.43-0.48)
Abbreviations: $PM_{2.5}$, ambient fine particulate matter; CI, confidence interval					

© 2019 Bowe B et al. *JAMA Network Open.*

eTable 4: Sensitivity analyses of the association between $PM_{2.5}$ and cause-specific mortality

**Cause Category**	**Baseline Exposure**	**Exposure by 3-year average prior to baseline**	**Time updated exposure[c]**	**Time updated cumulative exposure[d]**	**Exposure by AMS within 30 miles[a]**	**Exposure by AMS within 10 miles[b]**	**Additionally adjusted for latitude and longitude**	**Additionally adjusted for ozone[e]**
Non-accidental	1.12 (1.11-1.12)	1.09 (1.08-1.10)	1.20 (1.19-1.21)	1.12 (1.11-1.13)	1.13 (1.12-1.14)	1.11 (1.01-1.12)	1.09 (1.08-1.10)	1.10 (1.10-1.12)
Non-communicable diseases	1.12 (1.11-1.12)	1.09 (1.08-1.10)	1.20 (1.19-1.21)	1.12 (1.11-1.13)	1.13 (1.12-1.14)	1.11 (1.09-1.12)	1.09 (1.08-1.10)	1.11 (1.10-1.12)
Cardiovascular disease	1.19 (1.18-1.21)	1.18 (1.16-1.19)	1.27 (1.25-1.30)	1.21 (1.18-1.23)	1.17 (1.15-1.19)	1.14 (1.11-1.17)	1.15 (1.13-1.17)	1.21 (1.19-1.23)
Cerebrovascular disease	1.23 (1.19-1.26)	1.18 (1.14-1.22)	1.33 (1.28-1.39)	1.23 (1.18-1.28)	1.25 (1.20-1.29)	1.25 (1.19-1.31)	1.18 (1.14-1.23)	1.21 (1.17-1.25)
Chronic Kidney Disease	1.29 (1.22-1.36)	1.22 (1.16-1.29)	1.30 (1.22-1.39)	1.21 (1.13-1.29)	1.19 (1.12-1.26)	1.13 (1.05-1.22)	1.14 (1.07-1.22)	1.22 (1.15-1.29)
Chronic obstructive pulmonary disease	1.07 (1.05-1.10)	1.04 (1.02-1.07)	1.19 (1.15-1.22)	1.09 (1.06-1.13)	1.06 (1.03-1.09)	1.05 (1.01-1.09)	1.03 (1.00-1.07)	1.08 (1.05-1.11)
Dementia	1.09 (1.06-1.12)	1.04 (1.01-1.07)	1.19 (1.15-1.23)	1.07 (1.04-1.11)	1.13 (1.09-1.16)	1.11 (1.06-1.15)	1.04 (1.00-1.07)	1.03 (1.00-1.06)
Diabetes (Type 2)	1.36 (1.27-1.46)	1.38 (1.28-1.48)	1.57 (1.44-1.71)	1.49 (1.37-1.62)	1.18 (1.09-1.28)	1.10 (0.99-1.23)	1.45 (1.34-1.57)	1.46 (1.35-1.57)
Hypertension	1.25 (1.20-1.30)	1.23 (1.17-1.28)	1.55 (1.47-1.64)	1.39 (1.32-1.47)	1.34 (1.27-1.41)	1.34 (1.26-1.42)	1.23 (1.17-1.30)	1.16 (1.11-1.22)
Lung cancer	1.11 (1.09-1.14)	1.08 (1.05-1.10)	1.17 (1.13-1.20)	1.09 (1.06-1.12)	1.09 (1.06-1.12)	1.09 (1.05-1.13)	1.11 (1.08-1.14)	1.09 (1.06-1.12)
Pneumonia	1.54 (1.47-1.61)	1.50 (1.43-1.57)	1.77 (1.166-1.86)	1.60 (1.51-1.70)	1.50 (1.42-1.58)	1.48 (1.37-1.59)	1.29 (1.22-1.37)	1.54 (1.47-1.63)

[a] Analyses included those within 30 miles of the nearest air monitoring station that measured $PM_{2.5}$ (N=3489595)
[b] Analyses included those within 10 miles of the nearest air monitoring station that measured $PM_{2.5}$ (N=2077164)
[c] Analyses included those with a history of $PM_{2.5}$ in the year prior to a time point in analysis during following up (N=4496047)
[d] Analyses included those with a history of $PM_{2.5}$ exposure three years prior to baseline (N=3022654)
[e] Analyses included those within 30 miles of the nearest air monitoring station that measured ozone (N=3558042)

© 2019 Bowe B et al. *JAMA Network Open.*

eTable 5: Cause specific death associated with $PM_{2.5}$ in the contiguous US and by state

eTable 5a: Death due to non-accidental causes associated with $PM_{2.5}$ in the contiguous US and by state

Location	$PM_{2.5}$ (µg/m^3)	PAF (%) (95% UI)	Number of deaths (95% UI)	Rate (per 100,000) (95% UI)	Age-standardized rate (per 100,000) (95% UI)
Contiguous US	10.1	7.8 (7.2-8.4)	197905.1 (183463.3-213644.9)	61.2 (56.7-66)	51.4 (47.7-55.5)
Alabama	11.6	8.6 (8-9.3)	4198.4 (3918.7-4524.5)	86.1 (80.4-92.8)	71.3 (66.6-76.9)
Arizona	9.5	7.4 (6.8-8)	3809.1 (3521.5-4120)	54.3 (50.2-58.7)	43.9 (40.6-47.5)

© 2019 Bowe B et al. *JAMA Network Open.*

Arkansas	10.3	8 (7.5-8.6)	2394.1 (2229-2592.3)	79.7 (74.2-86.3)	65.1 (60.6-70.5)
California	12.2	8.7 (8.1-9.4)	21469.6 (19996.9-23161.1)	54.3 (50.6-58.6)	49.3 (45.9-53.2)
Colorado	7.3	6 (5.4-6.5)	1996.3 (1814.2-2167.2)	35.6 (32.4-38.7)	34.8 (31.6-37.7)
Connecticut	8.5	7 (6.4-7.5)	1997.9 (1844.1-2162)	55.7 (51.4-60.3)	40.6 (37.5-44)
Delaware	9.7	7.7 (7.2-8.3)	645.3 (596.2-699.1)	67.1 (62-72.7)	51.4 (47.5-55.7)
District of Columbia	9.7	7.7 (7.2-8.3)	335.1 (309.9-363.4)	48.3 (44.7-52.4)	49.2 (45.5-53.4)
Florida	7.6	6.3 (5.7-6.8)	11662.3 (10630.2-12615.4)	55.6 (50.7-60.1)	37.3 (34-40.4)
Georgia	11.0	8.3 (7.8-9)	6316.1 (5889.1-6818)	60.6 (56.5-65.4)	60.3 (56.3-65.1)
Idaho	9.2	7.3 (6.8-7.9)	923.8 (855.2-1003.1)	53.8 (49.8-58.4)	48.4 (44.8-52.6)
Illinois	13.3	9.2 (8.6-9.9)	9263 (8648.4-10001.8)	72.4 (67.6-78.1)	60.4 (56.4-65.2)
Indiana	12.2	8.8 (8.2-9.5)	5280.3 (4932-5695.4)	79.2 (74-85.4)	67.3 (62.9-72.6)
Iowa	9.3	7.5 (6.9-8.1)	2118.1 (1962.5-2286)	67.3 (62.4-72.7)	50.3 (46.6-54.3)
Kansas	9.4	7.5 (7-8.1)	1853.7 (1715.6-1999.4)	63.6 (58.9-68.6)	52.1 (48.2-56.2)
Kentucky	11.2	8.4 (7.9-9.1)	3678.4 (3433.7-3978.8)	82.6 (77.1-89.3)	69.8 (65.2-75.5)
Louisiana	9.2	7.5 (6.9-8.1)	3095 (2871.9-3352.1)	66.1 (61.3-71.6)	58.9 (54.6-63.8)
Maine	7.5	6.3 (5.8-6.8)	842.2 (768.5-912.6)	63 (57.5-68.3)	42.9 (39.1-46.5)
Maryland	10.3	8 (7.5-8.6)	3573 (3323.7-3849.4)	59 (54.9-63.6)	50.8 (47.2-54.7)
Massachusetts	7.9	6.5 (6-7.1)	3536.7 (3243.4-3828.4)	51.6 (47.3-55.8)	40.1 (36.8-43.4)
Michigan	10.7	8.1 (7.6-8.8)	7270.3 (6775.5-7843.3)	73 (68-78.7)	57.3 (53.4-61.8)
Minnesota	7.9	6.6 (6-7.1)	2655.3 (2431.6-2879.6)	47.6 (43.6-51.6)	38.9 (35.6-42.2)
Mississippi	10.1	7.9 (7.4-8.5)	2344.4 (2186.6-2538.3)	78.6 (73.3-85.1)	68.3 (63.7-74)
Missouri	10.6	8.2 (7.6-8.8)	4578.1 (4253.3-4936.4)	74.9 (69.6-80.7)	59.5 (55.2-64.1)
Montana	7.1	5.8 (5.3-6.3)	536.4 (484.5-579.8)	51.1 (46.1-55.2)	39 (35.2-42.1)
Nebraska	8.6	7 (6.5-7.6)	1099.2 (1010-1188.3)	57.2 (52.6-61.9)	46.8 (43-50.5)
Nevada	9.0	7.3 (6.7-7.8)	1611.8 (1490.1-1740.2)	53.8 (49.7-58)	49.8 (46.1-53.8)
New Hampshire	7.5	6.3 (5.8-6.8)	711.7 (647.1-772.1)	53 (48.2-57.5)	39.9 (36.2-43.3)
New Jersey	9.9	7.8 (7.2-8.4)	5381.7 (5004.2-5802.8)	59.8 (55.6-64.4)	47.2 (43.9-50.9)
New Mexico	6.7	5.6 (5-6)	916.5 (822.5-992.7)	43.9 (39.4-47.5)	36.2 (32.5-39.2)
New York	9.4	7.5 (7-8.1)	10919.4 (10127.4-11785.3)	55 (51-59.4)	43.3 (40.2-46.7)

© 2019 Bowe B et al. *JAMA Network Open.*

North Carolina	10.3	8 (7.4-8.6)	6773.4 (6307.3-7321.8)	65.9 (61.4-71.3)	56.4 (52.6-61)
North Dakota	6.0	4.8 (4.3-5.2)	284.7 (251.4-309.6)	37.7 (33.3-41)	30.3 (26.8-33)
Ohio	12.0	8.7 (8.2-9.4)	9769 (9122.8-10551.7)	83.8 (78.2-90.5)	65.5 (61.2-70.8)
Oklahoma	9.6	7.7 (7.1-8.3)	2814 (2619.8-3044.1)	71.6 (66.6-77.4)	62.1 (57.8-67.1)
Oregon	8.6	7.1 (6.5-7.6)	2362 (2174.9-2555.1)	57 (52.5-61.7)	45.7 (42.1-49.4)
Pennsylvania	10.9	8.2 (7.7-8.9)	10132.1 (9422.4-10933.4)	79.1 (73.6-85.4)	56.3 (52.3-60.7)
Rhode Island	7.1	6 (5.4-6.5)	553.1 (501.8-600.4)	52.2 (47.4-56.7)	38.1 (34.5-41.3)
South Carolina	10.0	7.9 (7.3-8.5)	3539 (3294-3822.8)	70.4 (65.6-76.1)	58.3 (54.3-63)
South Dakota	6.6	5.4 (4.8-5.8)	387.8 (346.3-421.1)	44.6 (39.8-48.4)	35 (31.3-38)
Tennessee	10.2	8 (7.4-8.6)	5083.7 (4728.6-5485)	75.7 (70.4-81.7)	64.3 (59.8-69.3)
Texas	10.3	8 (7.4-8.6)	14508.4 (13486.9-15653.8)	51.3 (47.6-55.3)	54 (50.2-58.3)
Utah	7.6	6.3 (5.8-6.8)	1005.3 (917.4-1085.3)	32.4 (29.6-35)	39.5 (36.1-42.7)
Vermont	6.9	5.7 (5.2-6.2)	312.5 (281.3-340.7)	50.1 (45.1-54.6)	36.3 (32.6-39.6)
Virginia	8.9	7.3 (6.7-7.9)	4580 (4236.2-4947.8)	54.1 (50-58.4)	47.7 (44.1-51.5)
Washington	7.9	6.5 (6-7.1)	3381 (3099.3-3659.7)	45.7 (41.9-49.4)	40.6 (37.2-43.9)
West Virginia	9.8	7.7 (7.2-8.4)	1609.9 (1499.2-1743.2)	88.7 (82.6-96)	63.9 (59.5-69.2)
Wisconsin	9.8	7.7 (7.1-8.3)	3652.8 (3389.6-3944.7)	63 (58.5-68.1)	49.2 (45.7-53.2)
Wyoming	4.7	3.4 (2.9-3.7)	143.1 (121.7-155.9)	24.7 (21-26.9)	21.1 (18-23)
Abbreviations: $PM_{2.5}$, ambient fine particulate matter; PAF, population attributable fraction; UI, uncertainty interval					

eTable 5b: Death due to non-communicable diseases associated with $PM_{2.5}$ in the contiguous US and by state

**Location**	**$PM_{2.5}$ ($\mu g/m^3$)**	**PAF (%) (95% UI)**	**Number of deaths (95% UI)**	**Rate (per 100,000) (95% UI)**	**Age-standardized rate (per 100,000) (95% UI)**
Contiguous US	10.1	7.7 (7.1-8.5)	188540.3 (173883.7-209786.3)	58.3 (53.7-64.8)	48.8 (45.1-54.3)
Alabama	11.6	8.5 (7.9-9.4)	3989.9 (3697.9-4453.5)	81.8 (75.9-91.4)	67.6 (62.6-75.4)
Arizona	9.5	7.3 (6.7-8.1)	3645 (3343.9-4090.8)	52 (47.7-58.3)	41.9 (38.4-47)
Arkansas	10.3	7.9 (7.3-8.8)	2287.5 (2122.5-2550.9)	76.1 (70.7-84.9)	62 (57.5-69.1)
California	12.2	8.6 (7.9-9.5)	20604.2 (19065.1-22898.3)	52.1 (48.2-57.9)	47.3 (43.7-52.5)

© 2019 Bowe B et al. *JAMA Network Open.*

Colorado	7.3	5.9 (5.3-6.6)	1903.9 (1716.5-2115.6)	34 (30.6-37.7)	33.1 (29.9-36.8)
Connecticut	8.5	6.9 (6.3-7.7)	1898.4 (1738-2117)	52.9 (48.4-59)	38.4 (35.2-42.8)
Delaware	9.7	7.6 (7-8.5)	616.3 (567.9-693.6)	64.1 (59-72.1)	48.9 (45.1-55.1)
District of Columbia	9.7	7.6 (7.1-8.5)	310.8 (286.8-349.2)	44.8 (41.3-50.3)	45.7 (42.1-51.3)
Florida	7.6	6.2 (5.6-6.9)	11153.1 (10093.9-12441.7)	53.1 (48.1-59.3)	35.4 (32-39.5)
Georgia	11.0	8.2 (7.6-9.1)	5964.2 (5537.2-6667.7)	57.2 (53.1-63.9)	57 (52.9-63.7)
Idaho	9.2	7.2 (6.6-8)	887.9 (814.3-989.2)	51.7 (47.4-57.6)	46.5 (42.6-51.8)
Illinois	13.3	9.1 (8.4-10.1)	8819.2 (8183.9-9809.2)	68.9 (63.9-76.6)	57.3 (53.2-63.8)
Indiana	12.2	8.7 (8.1-9.7)	5004.2 (4637.2-5568.5)	75.1 (69.6-83.5)	63.6 (58.9-70.8)
Iowa	9.3	7.4 (6.8-8.2)	2035.4 (1876.6-2279.4)	64.7 (59.7-72.5)	48.2 (44.4-54)
Kansas	9.4	7.4 (6.8-8.3)	1773.9 (1633.3-1984.9)	60.9 (56.1-68.1)	49.7 (45.8-55.6)
Kentucky	11.2	8.3 (7.7-9.3)	3485 (3227-3906.3)	78.2 (72.4-87.7)	66 (61.1-74)
Louisiana	9.2	7.4 (6.8-8.2)	2918.1 (2690.2-3252)	62.3 (57.4-69.4)	55.4 (51.1-61.8)
Maine	7.5	6.2 (5.7-7)	812.1 (736-904.5)	60.8 (55.1-67.7)	41.1 (37.3-45.8)
Maryland	10.3	7.9 (7.3-8.8)	3372.1 (3123.8-3756.9)	55.7 (51.6-62.1)	47.8 (44.3-53.2)
Massachusetts	7.9	6.5 (5.9-7.2)	3381.9 (3074-3773.3)	49.3 (44.8-55)	38.2 (34.7-42.6)
Michigan	10.7	8 (7.4-8.9)	6963.3 (6449.2-7751.7)	69.9 (64.7-77.8)	54.7 (50.6-60.9)
Minnesota	7.9	6.5 (5.9-7.2)	2550.6 (2321.1-2831.6)	45.7 (41.6-50.8)	37.2 (33.9-41.3)
Mississippi	10.1	7.8 (7.2-8.7)	2223.5 (2055.1-2479.8)	74.5 (68.9-83.1)	64.6 (59.7-72.1)
Missouri	10.6	8.1 (7.5-9)	4377.8 (4060.6-4865.2)	71.6 (66.4-79.6)	56.7 (52.6-63)
Montana	7.1	5.7 (5.2-6.4)	514.4 (461.7-579.7)	49 (44-55.2)	37.3 (33.5-42)
Nebraska	8.6	6.9 (6.3-7.7)	1055 (963.8-1165.6)	54.9 (50.2-60.7)	44.8 (40.9-49.5)
Nevada	9.0	7.2 (6.6-8)	1546 (1420.7-1712.3)	51.6 (47.4-57.1)	47.7 (43.9-52.9)
New Hampshire	7.5	6.2 (5.7-7)	683.3 (619.7-758.6)	50.9 (46.2-56.5)	38.1 (34.6-42.3)
New Jersey	9.9	7.7 (7.1-8.6)	5074.9 (4692.5-5642.3)	56.4 (52.1-62.7)	44.4 (41-49.3)
New Mexico	6.7	5.5 (4.9-6.1)	874.8 (781.1-975.3)	41.9 (37.4-46.7)	34.5 (30.8-38.4)
New York	9.4	7.4 (6.9-8.3)	10410 (9603.9-11620.1)	52.4 (48.4-58.5)	41.1 (38-45.9)
North Carolina	10.3	7.9 (7.3-8.8)	6429.4 (5952.9-7171.8)	62.6 (57.9-69.8)	53.4 (49.5-59.6)
North Dakota	6.0	4.8 (4.2-5.3)	273 (238-307.6)	36.1 (31.5-40.7)	29 (25.3-32.7)

© 2019 Bowe B et al. *JAMA Network Open.*

Ohio	12.0	8.6 (8-9.6)	9307.9 (8646.2-10368.1)	79.8 (74.2-88.9)	62.2 (57.7-69.2)
Oklahoma	9.6	7.6 (7-8.4)	2689.7 (2483.9-3008.7)	68.4 (63.2-76.5)	59.2 (54.7-66.2)
Oregon	8.6	7 (6.4-7.8)	2266.7 (2084-2523.3)	54.7 (50.3-60.9)	43.7 (40.2-48.7)
Pennsylvania	10.9	8.1 (7.5-9.1)	9647.6 (8919.4-10749.5)	75.3 (69.7-83.9)	53.3 (49.3-59.4)
Rhode Island	7.1	5.9 (5.3-6.6)	531.1 (477.2-593.9)	50.1 (45-56)	36.3 (32.6-40.6)
South Carolina	10.0	7.8 (7.2-8.7)	3359.8 (3107.9-3757.1)	66.9 (61.9-74.8)	55.2 (51.1-61.7)
South Dakota	6.6	5.3 (4.7-5.9)	369.3 (330.2-407.5)	42.5 (38-46.9)	33.2 (29.7-36.6)
Tennessee	10.2	7.9 (7.3-8.8)	4846.1 (4485.3-5417.3)	72.2 (66.8-80.7)	61.1 (56.5-68.3)
Texas	10.3	7.9 (7.3-8.8)	13652.6 (12615.5-15162.3)	48.2 (44.6-53.6)	51 (47.1-56.6)
Utah	7.6	6.2 (5.7-7)	951 (860.6-1068.2)	30.7 (27.7-34.4)	37.6 (34-42.2)
Vermont	6.9	5.7 (5.1-6.3)	301.7 (267.8-337.2)	48.4 (42.9-54.1)	34.9 (31-39)
Virginia	8.9	7.2 (6.6-8)	4347.2 (3988.1-4857.5)	51.3 (47.1-57.3)	45.2 (41.5-50.5)
Washington	7.9	6.5 (5.9-7.2)	3244.8 (2953.3-3609.5)	43.8 (39.9-48.7)	38.9 (35.4-43.3)
West Virginia	9.8	7.6 (7.1-8.5)	1539.5 (1426.7-1696.5)	84.8 (78.6-93.4)	60.8 (56.3-67)
Wisconsin	9.8	7.6 (7-8.4)	3508.9 (3237.2-3903.3)	60.5 (55.9-67.4)	47.1 (43.4-52.4)
Wyoming	4.7	3.3 (2.8-3.7)	137.2 (116.4-153.3)	23.7 (20.1-26.5)	20.2 (17.2-22.6)
Abbreviations: $PM_{2.5}$, ambient fine particulate matter; PAF, population attributable fraction; UI, uncertainty interval					

eTable 5c: Death due to cardiovascular disease associated with $PM_{2.5}$ in the contiguous US and by state

**Location**	**$PM_{2.5}$ (μg/m^3)**	**PAF (%) (95% UI)**	**Number of deaths (95% UI)**	**Rate (per 100,000) (95% UI)**	**Age-standardized rate (per 100,000) (95% UI)**
Contiguous US	10.1	12.6 (11.7-13.5)	56070.1 (51940.2-60318.3)	17.3 (16.1-18.6)	14.4 (13.3-15.5)
Alabama	11.6	14.9 (13.8-16)	1113.4 (1020.5-1202.1)	22.8 (20.9-24.7)	18.9 (17.3-20.4)
Arizona	9.5	11.7 (10.8-12.5)	1012.2 (932.5-1093.5)	14.4 (13.3-15.6)	11.5 (10.6-12.4)
Arkansas	10.3	12.9 (12-13.9)	794.4 (732.8-858.8)	26.4 (24.4-28.6)	21.3 (19.7-23)

© 2019 Bowe B et al. *JAMA Network Open.*

California	12.2	15.7 (14.6-16.8)	7119.3 (6601.7-7659)	18 (16.7-19.4)	16.2 (15-17.4)
Colorado	7.3	8.3 (7.6-8.9)	367.6 (337.3-398.8)	6.6 (6-7.1)	6.4 (5.8-6.9)
Connecticut	8.5	10.2 (9.5-11)	488.3 (446.7-530)	13.6 (12.5-14.8)	9.6 (8.8-10.5)
Delaware	9.7	12 (11.2-13)	158 (142.7-172.4)	16.4 (14.8-17.9)	12.4 (11.2-13.5)
District of Columbia	9.7	12.1 (11.2-13)	93.3 (83.1-103.3)	13.4 (12-14.9)	13.9 (12.3-15.4)
Florida	7.6	8.7 (8.1-9.4)	2900.8 (2671.3-3137.5)	13.8 (12.7-15)	9 (8.3-9.7)
Georgia	11.0	14 (12.9-15)	1553.7 (1436.3-1680.4)	14.9 (13.8-16.1)	15 (13.8-16.2)
Idaho	9.2	11.3 (10.5-12.2)	232.8 (210.2-253)	13.6 (12.2-14.7)	12.2 (11-13.3)
Illinois	13.3	17.4 (16.1-18.6)	2953.9 (2712.2-3178.9)	23.1 (21.2-24.8)	19 (17.4-20.4)
Indiana	12.2	15.8 (14.7-17)	1581.3 (1458.8-1713)	23.7 (21.9-25.7)	20 (18.4-21.6)
Iowa	9.3	11.4 (10.6-12.3)	577.5 (529.6-623.4)	18.4 (16.8-19.8)	13.4 (12.3-14.5)
Kansas	9.4	11.6 (10.7-12.5)	473.2 (435-512.1)	16.2 (14.9-17.6)	13 (12-14.1)
Kentucky	11.2	14.4 (13.3-15.4)	1004.1 (926.1-1085.8)	22.5 (20.8-24.4)	18.9 (17.5-20.5)
Louisiana	9.2	11.4 (10.5-12.3)	830.7 (759.5-897.9)	17.7 (16.2-19.2)	15.8 (14.4-17)
Maine	7.5	8.7 (8-9.4)	166.6 (152.1-182.4)	12.5 (11.4-13.7)	8.3 (7.6-9.1)
Maryland	10.3	12.9 (12-13.9)	974.1 (895.2-1055.3)	16.1 (14.8-17.4)	13.7 (12.6-14.9)
Massachusetts	7.9	9.2 (8.5-9.9)	756.5 (694.9-818.4)	11 (10.1-11.9)	8.4 (7.7-9)
Michigan	10.7	13.5 (12.5-14.5)	2498.2 (2313.6-2693.4)	25.1 (23.2-27)	19.3 (17.9-20.9)
Minnesota	7.9	9.3 (8.6-10)	494 (452.3-535.2)	8.9 (8.1-9.6)	7.2 (6.5-7.7)
Mississippi	10.1	12.7 (11.7-13.6)	649.2 (595.5-702.8)	21.8 (20-23.6)	18.9 (17.3-20.4)
Missouri	10.6	13.5 (12.5-14.5)	1429.2 (1321.3-1536.6)	23.4 (21.6-25.1)	18.4 (17-19.7)
Montana	7.1	7.9 (7.3-8.5)	122.2 (110.9-133.4)	11.6 (10.6-12.7)	8.7 (7.9-9.5)
Nebraska	8.6	10.4 (9.6-11.2)	243.2 (222.1-264.6)	12.7 (11.6-13.8)	10.1 (9.2-11)
Nevada	9.0	10.9 (10.1-11.8)	432.2 (395.5-468.7)	14.4 (13.2-15.6)	13.4 (12.3-14.6)
New Hampshire	7.5	8.7 (8-9.4)	163.2 (147.9-179)	12.2 (11-13.3)	9 (8.1-9.8)
New Jersey	9.9	12.4 (11.4-13.3)	1600.4 (1476.4-1729.6)	17.8 (16.4-19.2)	13.7 (12.7-14.8)
New Mexico	6.7	7.4 (6.8-7.9)	223.6 (204.4-243.9)	10.7 (9.8-11.7)	8.6 (7.9-9.4)
New York	9.4	11.7 (10.8-12.6)	3858.8 (3571.7-4162.7)	19.4 (18-21)	14.9 (13.8-16.1)
North Carolina	10.3	12.9 (12-13.9)	1690.6 (1557.5-1828.5)	16.5 (15.2-17.8)	14 (12.9-15.1)

© 2019 Bowe B et al. *JAMA Network Open.*

North Dakota	6.0	6.1 (5.7-6.6)	57.9 (51.9-63.7)	7.7 (6.9-8.4)	6 (5.4-6.6)
Ohio	12.0	15.5 (14.3-16.6)	2940.2 (2720.1-3179.3)	25.2 (23.3-27.3)	19.4 (18-21)
Oklahoma	9.6	12 (11.1-12.9)	807.1 (744.6-875.6)	20.5 (18.9-22.3)	17.7 (16.3-19.2)
Oregon	8.6	10.4 (9.6-11.2)	459.3 (419.9-498.2)	11.1 (10.1-12)	8.8 (8.1-9.6)
Pennsylvania	10.9	13.8 (12.8-14.8)	3100.1 (2872.8-3340.5)	24.2 (22.4-26.1)	16.8 (15.5-18.1)
Rhode Island	7.1	8 (7.4-8.7)	141.3 (127.8-154.4)	13.3 (12.1-14.6)	9.4 (8.5-10.2)
South Carolina	10.0	12.6 (11.6-13.5)	849.8 (779.2-920.4)	16.9 (15.5-18.3)	13.9 (12.7-15.1)
South Dakota	6.6	7.1 (6.6-7.7)	83.8 (75.2-92.4)	9.6 (8.7-10.6)	7.3 (6.6-8.1)
Tennessee	10.2	12.9 (11.9-13.9)	1455.4 (1342.4-1570.7)	21.7 (20-23.4)	18.2 (16.8-19.7)
Texas	10.3	13 (12.1-14)	4051.2 (3736.4-4366.2)	14.3 (13.2-15.4)	15.1 (13.9-16.3)
Utah	7.6	8.9 (8.2-9.5)	231.1 (212.1-251.4)	7.5 (6.8-8.1)	9.3 (8.5-10.1)
Vermont	6.9	7.6 (7.1-8.2)	68.4 (61.3-75.2)	11 (9.8-12.1)	7.8 (7-8.5)
Virginia	8.9	10.9 (10.1-11.8)	1096.2 (1007.6-1185.3)	12.9 (11.9-14)	11.3 (10.4-12.3)
Washington	7.9	9.3 (8.6-10)	689.4 (632.7-747.8)	9.3 (8.5-10.1)	8.2 (7.5-8.9)
West Virginia	9.8	12.2 (11.3-13.1)	460.1 (419.9-498.8)	25.3 (23.1-27.5)	18.1 (16.5-19.6)
Wisconsin	9.8	12.1 (11.2-13.1)	994.2 (916.4-1078)	17.2 (15.8-18.6)	13.2 (12.1-14.3)
Wyoming	4.7	4.1 (3.8-4.4)	28.2 (25.2-31.6)	4.9 (4.4-5.4)	4.2 (3.7-4.7)
Abbreviations: $PM_{2.5}$, ambient fine particulate matter; PAF, population attributable fraction; UI, uncertainty interval					

eTable 5d: Death due to cerebrovascular disease associated with $PM_{2.5}$ in the contiguous US and by state

Location	$PM_{2.5}$ (μg/m^3)	PAF (%) (95% UI)	Number of deaths (95% UI)	Rate (per 100,000) (95% UI)	Age-standardized rate (per 100,000) (95% UI)
Contiguous US	10.1	27.8 (15-31.9)	40466.1 (21770.1-46487.9)	12.5 (6.7-14.4)	10.6 (5.7-12.1)
Alabama	11.6	30.3 (16.9-34.8)	888.7 (498.4-1023.4)	18.2 (10.2-21)	15.2 (8.5-17.5)
Arizona	9.5	26.7 (14.3-30.7)	714.5 (376.1-824.8)	10.2 (5.4-11.8)	8.2 (4.3-9.5)

© 2019 Bowe B et al. *JAMA Network Open.*

Arkansas	10.3	28.5 (15.4-32.8)	460.3 (249.8-532.7)	15.3 (8.3-17.7)	12.5 (6.8-14.5)
California	12.2	30.7 (17.6-35.2)	5024.4 (2883.8-5734.9)	12.7 (7.3-14.5)	11.5 (6.6-13.2)
Colorado	7.3	22.3 (11.5-25.8)	443.7 (228-511.9)	7.9 (4.1-9.1)	8 (4.1-9.2)
Connecticut	8.5	25.4 (13.5-29.2)	355.7 (187.8-412.1)	9.9 (5.2-11.5)	7 (3.7-8.2)
Delaware	9.7	27.6 (14.9-31.7)	157.7 (84.1-185)	16.4 (8.7-19.2)	12.8 (6.8-15)
District of Columbia	9.7	27.6 (14.9-31.8)	67.9 (36.2-81.9)	9.8 (5.2-11.8)	9.9 (5.3-12)
Florida	7.6	23.3 (12.1-26.9)	2936.6 (1529.2-3396.1)	14 (7.3-16.2)	9.1 (4.7-10.5)
Georgia	11.0	29.5 (16.1-33.9)	1299.4 (718-1492.9)	12.5 (6.9-14.3)	12.8 (7.1-14.8)
Idaho	9.2	26.4 (14.1-30.4)	191.8 (100.8-223.4)	11.2 (5.9-13)	10.2 (5.4-11.8)
Illinois	13.3	32.3 (19.3-37)	1944.7 (1146.9-2232.4)	15.2 (9-17.4)	12.6 (7.4-14.4)
Indiana	12.2	31.2 (17.9-35.7)	980.6 (557.3-1129)	14.7 (8.4-16.9)	12.5 (7.1-14.4)
Iowa	9.3	26.9 (14.4-30.9)	380.8 (198.6-443.2)	12.1 (6.3-14.1)	8.8 (4.6-10.3)
Kansas	9.4	27 (14.5-31)	365.7 (192.6-427.9)	12.6 (6.6-14.7)	10.2 (5.4-11.9)
Kentucky	11.2	29.8 (16.4-34.2)	611.5 (345.9-704.1)	13.7 (7.8-15.8)	11.8 (6.7-13.5)
Louisiana	9.2	26.8 (14.4-30.8)	659.8 (351.9-762.3)	14.1 (7.5-16.3)	12.7 (6.8-14.7)
Maine	7.5	23.4 (12.1-27)	172 (89-202.6)	12.9 (6.7-15.2)	8.8 (4.5-10.3)
Maryland	10.3	28.5 (15.4-32.7)	804.3 (442.1-929.8)	13.3 (7.3-15.4)	11.5 (6.3-13.3)
Massachusetts	7.9	24.1 (12.6-27.7)	569.5 (294.6-663.6)	8.3 (4.3-9.7)	6.4 (3.3-7.5)
Michigan	10.7	28.9 (15.8-33.2)	1447.6 (791.2-1663.9)	14.5 (7.9-16.7)	11.4 (6.2-13.1)
Minnesota	7.9	24.1 (12.6-27.8)	541.3 (283.4-629)	9.7 (5.1-11.3)	7.9 (4.1-9.1)
Mississippi	10.1	28.2 (15.2-32.4)	486.2 (259.7-561.8)	16.3 (8.7-18.8)	14.4 (7.7-16.7)
Missouri	10.6	29 (15.8-33.3)	916.5 (504.1-1053.7)	15 (8.2-17.2)	11.9 (6.5-13.7)
Montana	7.1	21.9 (11.2-25.3)	106.5 (55.3-126.2)	10.1 (5.3-12)	7.8 (4-9.2)
Nebraska	8.6	25.4 (13.6-29.3)	193.4 (99.2-224.9)	10.1 (5.2-11.7)	8 (4.1-9.3)
Nevada	9.0	26.2 (14-30.2)	297.9 (161-346.2)	9.9 (5.4-11.5)	9.4 (5.1-10.9)
New Hampshire	7.5	23.4 (12.1-27)	120.1 (61.9-142.7)	8.9 (4.6-10.6)	6.8 (3.5-8)
New Jersey	9.9	27.9 (15-32)	969.3 (512.4-1117.7)	10.8 (5.7-12.4)	8.4 (4.4-9.7)
New Mexico	6.7	21.1 (10.8-24.5)	185.4 (95.9-217.9)	8.9 (4.6-10.4)	7.3 (3.8-8.6)
New York	9.4	27.1 (14.5-31.1)	1698.5 (901.5-1962.3)	8.6 (4.5-9.9)	6.7 (3.5-7.7)

© 2019 Bowe B et al. *JAMA Network Open.*

North Carolina	10.3	28.5 (15.4-32.7)	1451.8 (804.9-1674.1)	14.1 (7.8-16.3)	12.3 (6.8-14.1)
North Dakota	6.0	18.9 (9.4-22)	63.7 (32-75.5)	8.4 (4.2-10)	6.7 (3.4-8)
Ohio	12.0	30.9 (17.5-35.4)	1982.2 (1111.6-2275.3)	17 (9.5-19.5)	13.2 (7.4-15.2)
Oklahoma	9.6	27.5 (14.8-31.6)	534.5 (292.7-615.5)	13.6 (7.4-15.7)	11.9 (6.5-13.7)
Oregon	8.6	25.6 (13.7-29.5)	529.4 (283.9-611.3)	12.8 (6.9-14.8)	10.2 (5.5-11.8)
Pennsylvania	10.9	29.3 (16-33.6)	1961.6 (1068.6-2257.1)	15.3 (8.3-17.6)	10.7 (5.8-12.3)
Rhode Island	7.1	22.3 (11.4-25.8)	94.9 (48.6-112.7)	9 (4.6-10.6)	6.6 (3.4-7.8)
South Carolina	10.0	28.1 (15.2-32.3)	757.6 (410.7-877.1)	15.1 (8.2-17.5)	12.6 (6.8-14.6)
South Dakota	6.6	20.5 (10.4-23.7)	84.8 (42.8-101.2)	9.8 (4.9-11.6)	7.5 (3.8-9)
Tennessee	10.2	28.5 (15.4-32.7)	1001.4 (540.7-1152.3)	14.9 (8.1-17.2)	12.8 (6.9-14.7)
Texas	10.3	28.5 (15.4-32.7)	3074.8 (1665.5-3534.6)	10.9 (5.9-12.5)	11.8 (6.4-13.5)
Utah	7.6	23.4 (12.2-26.9)	207.2 (108.9-243.4)	6.7 (3.5-7.8)	8.5 (4.4-9.9)
Vermont	6.9	21.6 (11-25)	53.7 (26-63.9)	8.6 (4.2-10.3)	6.2 (3-7.4)
Virginia	8.9	26.3 (14.1-30.2)	935.5 (494-1081)	11 (5.8-12.8)	9.9 (5.2-11.4)
Washington	7.9	24.1 (12.6-27.7)	728.8 (377.3-846.1)	9.8 (5.1-11.4)	8.9 (4.6-10.3)
West Virginia	9.8	27.7 (14.9-31.8)	293.2 (154.8-343.3)	16.1 (8.5-18.9)	11.6 (6.1-13.6)
Wisconsin	9.8	27.5 (14.8-31.6)	691.5 (368.6-793.7)	11.9 (6.4-13.7)	9.2 (4.9-10.6)
Wyoming	4.7	14.1 (6.7-16.5)	26.9 (12.1-33.3)	4.6 (2.1-5.7)	4 (1.8-5)
Abbreviations: $PM_{2.5}$, ambient fine particulate matter; PAF, population attributable fraction; UI, uncertainty interval					

eTable 5e: Death due to chronic kidney disease associated with $PM_{2.5}$ in the contiguous US and by state

**Location**	**$PM_{2.5}$ (μg/m^3)**	**PAF (%) (95% UI)**	**Number of deaths (95% UI)**	**Rate (per 100,000) (95% UI)**	**Age-standardized rate (per 100,000) (95% UI)**
Contiguous US	10.1	17.1 (14-19.9)	7175.2 (5910.2-8371.9)	2.2 (1.8-2.6)	1.9 (1.5-2.2)
Alabama	11.6	19.3 (16.2-22.4)	157.4 (129.2-184.3)	3.2 (2.6-3.8)	2.6 (2.2-3.1)

© 2019 Bowe B et al. *JAMA Network Open.*

Arizona	9.5	16.2 (13.1-18.9)	70.3 (55.5-83.7)	1 (0.8-1.2)	0.8 (0.6-1)
Arkansas	10.3	17.9 (14.9-20.8)	105.4 (85.4-125.4)	3.5 (2.8-4.2)	2.9 (2.3-3.4)
California	12.2	19.4 (16.2-22.5)	690.7 (575.2-804.8)	1.7 (1.5-2)	1.6 (1.3-1.8)
Colorado	7.3	12.2 (9.3-14.7)	49.9 (36.9-61.8)	0.9 (0.7-1.1)	0.9 (0.6-1.1)
Connecticut	8.5	15 (11.9-17.7)	71 (55.2-85.4)	2 (1.5-2.4)	1.4 (1.1-1.7)
Delaware	9.7	17.1 (14-19.9)	30.1 (23.2-36.9)	3.1 (2.4-3.8)	2.4 (1.8-2.9)
District of Columbia	9.7	17.2 (14-20)	10 (7-13.2)	1.4 (1-1.9)	1.5 (1-2)
Florida	7.6	13 (9.9-15.6)	332.7 (254.1-402.4)	1.6 (1.2-1.9)	1.1 (0.8-1.3)
Georgia	11.0	18.7 (15.6-21.7)	304.5 (251.6-358.5)	2.9 (2.4-3.4)	2.9 (2.4-3.4)
Idaho	9.2	15.9 (12.8-18.6)	22.6 (17.3-28.3)	1.3 (1-1.6)	1.2 (0.9-1.5)
Illinois	13.3	20.7 (17.5-23.9)	443.2 (369.5-513.8)	3.5 (2.9-4)	2.9 (2.4-3.4)
Indiana	12.2	20 (16.7-23)	227.5 (189.7-262.7)	3.4 (2.8-3.9)	2.9 (2.4-3.4)
Iowa	9.3	16.5 (13.3-19.2)	51.3 (40-63.2)	1.6 (1.3-2)	1.2 (1-1.5)
Kansas	9.4	16.5 (13.4-19.3)	71.8 (56.1-85.7)	2.5 (1.9-2.9)	2 (1.6-2.4)
Kentucky	11.2	18.9 (15.8-21.9)	145.1 (119.1-168.9)	3.3 (2.7-3.8)	2.8 (2.3-3.2)
Louisiana	9.2	16.4 (13.3-19.2)	149.1 (120.4-175.9)	3.2 (2.6-3.8)	2.9 (2.3-3.4)
Maine	7.5	13.1 (10-15.7)	30.3 (22.4-37.6)	2.3 (1.7-2.8)	1.5 (1.1-1.9)
Maryland	10.3	17.9 (14.8-20.8)	125.6 (101.6-146.8)	2.1 (1.7-2.4)	1.8 (1.4-2.1)
Massachusetts	7.9	13.8 (10.6-16.4)	137.9 (103.9-167.4)	2 (1.5-2.4)	1.6 (1.2-1.9)
Michigan	10.7	18.2 (15.2-21.1)	284.4 (234.7-331.7)	2.9 (2.4-3.3)	2.2 (1.8-2.6)
Minnesota	7.9	13.8 (10.7-16.4)	64.6 (49.3-79.3)	1.2 (0.9-1.4)	0.9 (0.7-1.1)
Mississippi	10.1	17.7 (14.6-20.5)	107.5 (86.3-127.7)	3.6 (2.9-4.3)	3.1 (2.5-3.7)
Missouri	10.6	18.3 (15.2-21.2)	214.4 (176.7-250.3)	3.5 (2.9-4.1)	2.8 (2.3-3.2)
Montana	7.1	11.7 (8.8-14.2)	12.4 (8.5-16.2)	1.2 (0.8-1.5)	0.9 (0.6-1.2)
Nebraska	8.6	15.1 (12.1-17.8)	27.9 (21.4-34.5)	1.5 (1.1-1.8)	1.2 (0.9-1.5)
Nevada	9.0	15.8 (12.8-18.6)	38.9 (30.2-47.6)	1.3 (1-1.6)	1.2 (0.9-1.5)
New Hampshire	7.5	13.1 (9.9-15.7)	18.1 (13.1-23.1)	1.3 (1-1.7)	1 (0.7-1.3)
New Jersey	9.9	17.3 (14.2-20.2)	223.1 (182.2-261.2)	2.5 (2-2.9)	2 (1.6-2.3)
New Mexico	6.7	11 (8-13.4)	28.9 (20.9-36.6)	1.4 (1-1.8)	1.1 (0.8-1.4)

© 2019 Bowe B et al. *JAMA Network Open.*

New York	9.4	16.6 (13.5-19.4)	312.8 (255-365.9)	1.6 (1.3-1.8)	1.2 (1-1.5)
North Carolina	10.3	17.8 (14.8-20.8)	298.9 (246.1-346.8)	2.9 (2.4-3.4)	2.5 (2-2.9)
North Dakota	6.0	9 (6.2-11.3)	9.5 (6.1-12.8)	1.3 (0.8-1.7)	1 (0.6-1.3)
Ohio	12.0	19.7 (16.5-22.8)	372.7 (307.2-430.7)	3.2 (2.6-3.7)	2.5 (2.1-2.9)
Oklahoma	9.6	17 (13.9-19.8)	64.7 (51.5-78.5)	1.6 (1.3-2)	1.4 (1.1-1.7)
Oregon	8.6	15.3 (12.1-17.9)	44.2 (34.3-52.9)	1.1 (0.8-1.3)	0.9 (0.7-1)
Pennsylvania	10.9	18.5 (15.4-21.5)	440.3 (361.3-511.8)	3.4 (2.8-4)	2.4 (2-2.8)
Rhode Island	7.1	12.1 (9-14.7)	18.6 (13.4-23.5)	1.8 (1.3-2.2)	1.2 (0.9-1.5)
South Carolina	10.0	17.6 (14.5-20.4)	133.2 (108.6-157.2)	2.7 (2.2-3.1)	2.2 (1.8-2.6)
South Dakota	6.6	10.5 (7.7-12.9)	6.9 (4.6-9.3)	0.8 (0.5-1.1)	0.6 (0.4-0.8)
Tennessee	10.2	17.8 (14.8-20.7)	162.9 (131.8-193.6)	2.4 (2-2.9)	2.1 (1.7-2.5)
Texas	10.3	17.8 (14.8-20.7)	565.3 (460.8-660.1)	2 (1.6-2.3)	2.1 (1.7-2.5)
Utah	7.6	13.2 (10.1-15.7)	43.7 (32.9-53.7)	1.4 (1.1-1.7)	1.7 (1.3-2.1)
Vermont	6.9	11.4 (8.4-13.9)	2.7 (1.5-4.1)	0.4 (0.2-0.7)	0.3 (0.2-0.5)
Virginia	8.9	15.9 (12.8-18.6)	211.6 (170.6-250.3)	2.5 (2-3)	2.2 (1.8-2.6)
Washington	7.9	13.7 (10.6-16.4)	47.8 (35.7-59.4)	0.6 (0.5-0.8)	0.6 (0.4-0.7)
West Virginia	9.8	17.2 (14.1-20)	62.4 (49.6-75)	3.4 (2.7-4.1)	2.4 (1.9-3)
Wisconsin	9.8	16.9 (13.8-19.7)	127.4 (102-150.2)	2.2 (1.8-2.6)	1.7 (1.4-2)
Wyoming	4.7	5.6 (3.6-7.4)	3 (1.7-4.5)	0.5 (0.3-0.8)	0.5 (0.3-0.7)
Abbreviations: $PM_{2.5}$, ambient fine particulate matter; PAF, population attributable fraction; UI, uncertainty interval					

eTable 5f: Death due to chronic obstructive pulmonary disease associated with $PM_{2.5}$ in the contiguous US and by state

Location	$PM_{2.5}$ (μg/m^3)	PAF (%) (95% UI)	Number of deaths (95% UI)	Rate (per 100,000) (95% UI)	Age-standardized rate (per 100,000) (95% UI)
Contiguous US	10.1	0.4 (0.2-1.6)	645.7 (300.2-2490.9)	0.2 (0.1-0.8)	0.2 (0.1-0.6)

© 2019 Bowe B et al. *JAMA Network Open.*

Alabama	11.6	0.6 (0.3-2.2)	20.6 (8.9-76.1)	0.4 (0.2-1.6)	0.3 (0.1-1.3)
Arizona	9.5	0.2 (0.1-1.3)	9 (3.7-49.5)	0.1 (0.1-0.7)	0.1 (0-0.6)
Arkansas	10.3	0.3 (0.1-1.5)	6.7 (2.7-38)	0.2 (0.1-1.3)	0.2 (0.1-1)
California	12.2	1.3 (0.7-2.9)	175.7 (94.5-387.1)	0.4 (0.2-1)	0.4 (0.2-0.9)
Colorado	7.3	0.1 (0-0.6)	1.5 (0.6-15.3)	0 (0-0.3)	0 (0-0.3)
Connecticut	8.5	0.1 (0-0.9)	1.6 (0.6-12.8)	0 (0-0.4)	0 (0-0.3)
Delaware	9.7	0.2 (0.1-1.3)	1 (0.4-6.6)	0.1 (0-0.7)	0.1 (0-0.5)
District of Columbia	9.7	0.2 (0.1-1.3)	0.2 (0.1-1.6)	0 (0-0.2)	0 (0-0.2)
Florida	7.6	0.1 (0-0.6)	8 (3.2-79.9)	0 (0-0.4)	0 (0-0.2)
Georgia	11.0	0.4 (0.2-1.9)	19.5 (8.1-88.6)	0.2 (0.1-0.8)	0.2 (0.1-0.8)
Idaho	9.2	0.2 (0.1-1.2)	2 (0.8-11)	0.1 (0-0.6)	0.1 (0-0.6)
Illinois	13.3	1.6 (0.8-3.5)	86.5 (42.5-191.5)	0.7 (0.3-1.5)	0.6 (0.3-1.3)
Indiana	12.2	0.8 (0.4-2.6)	35.9 (15.9-112.1)	0.5 (0.2-1.7)	0.5 (0.2-1.4)
Iowa	9.3	0.2 (0.1-1.1)	3 (1.2-21.6)	0.1 (0-0.7)	0.1 (0-0.5)
Kansas	9.4	0.2 (0.1-1.2)	3.1 (1.2-20.9)	0.1 (0-0.7)	0.1 (0-0.6)
Kentucky	11.2	0.5 (0.2-2.1)	18.1 (7.7-70.5)	0.4 (0.2-1.6)	0.3 (0.1-1.3)
Louisiana	9.2	0.2 (0.1-1.1)	3.7 (1.5-26.7)	0.1 (0-0.6)	0.1 (0-0.5)
Maine	7.5	0.1 (0-0.6)	0.5 (0.2-5.9)	0 (0-0.4)	0 (0-0.3)
Maryland	10.3	0.3 (0.1-1.5)	5.6 (2.3-30.6)	0.1 (0-0.5)	0.1 (0-0.4)
Massachusetts	7.9	0.1 (0-0.7)	1.8 (0.7-19)	0 (0-0.3)	0 (0-0.2)
Michigan	10.7	0.4 (0.2-1.8)	21.2 (8.8-98.2)	0.2 (0.1-1)	0.2 (0.1-0.8)
Minnesota	7.9	0.1 (0-0.7)	1.7 (0.7-17)	0 (0-0.3)	0 (0-0.3)
Mississippi	10.1	0.2 (0.1-1.4)	4.8 (1.9-28.9)	0.2 (0.1-1)	0.1 (0.1-0.8)
Missouri	10.6	0.4 (0.2-1.7)	14.1 (5.8-66.5)	0.2 (0.1-1.1)	0.2 (0.1-0.9)
Montana	7.1	0.1 (0-0.6)	0.4 (0.1-4)	0 (0-0.4)	0 (0-0.3)
Nebraska	8.6	0.1 (0-0.9)	1.4 (0.6-11.2)	0.1 (0-0.6)	0.1 (0-0.5)
Nevada	9.0	0.1 (0.1-1)	2.2 (0.9-16.6)	0.1 (0-0.6)	0.1 (0-0.5)
New Hampshire	7.5	0.1 (0-0.6)	0.4 (0.2-4.5)	0 (0-0.3)	0 (0-0.3)
New Jersey	9.9	0.2 (0.1-1.4)	7.3 (3-42.4)	0.1 (0-0.5)	0.1 (0-0.4)

© 2019 Bowe B et al. *JAMA Network Open.*

New Mexico	6.7	0 (0-0.4)	0.4 (0.1-4.9)	0 (0-0.2)	0 (0-0.2)
New York	9.4	0.2 (0.1-1.2)	13.1 (5.3-84.2)	0.1 (0-0.4)	0.1 (0-0.3)
North Carolina	10.3	0.3 (0.1-1.6)	15.6 (6.4-83.9)	0.2 (0.1-0.8)	0.1 (0.1-0.7)
North Dakota	6.0	0 (0-0.3)	0.1 (0-1.1)	0 (0-0.1)	0 (0-0.1)
Ohio	12.0	0.7 (0.3-2.4)	51.3 (22.1-176.6)	0.4 (0.2-1.5)	0.3 (0.1-1.2)
Oklahoma	9.6	0.2 (0.1-1.3)	5.6 (2.3-37.1)	0.1 (0.1-0.9)	0.1 (0-0.8)
Oregon	8.6	0.1 (0-0.9)	2.2 (0.9-18.4)	0.1 (0-0.4)	0 (0-0.3)
Pennsylvania	10.9	0.4 (0.2-1.9)	28.5 (12.2-121.9)	0.2 (0.1-1)	0.2 (0.1-0.7)
Rhode Island	7.1	0 (0-0.5)	0.2 (0.1-2.6)	0 (0-0.2)	0 (0-0.2)
South Carolina	10.0	0.2 (0.1-1.4)	7 (2.9-40.7)	0.1 (0.1-0.8)	0.1 (0-0.7)
South Dakota	6.6	0 (0-0.5)	0.2 (0.1-2.2)	0 (0-0.2)	0 (0-0.2)
Tennessee	10.2	0.3 (0.1-1.5)	12.3 (5.1-69)	0.2 (0.1-1)	0.2 (0.1-0.8)
Texas	10.3	0.3 (0.1-1.6)	33.6 (14-166.9)	0.1 (0-0.6)	0.1 (0.1-0.6)
Utah	7.6	0.1 (0-0.7)	0.5 (0.2-5.3)	0 (0-0.2)	0 (0-0.2)
Vermont	6.9	0 (0-0.5)	0.1 (0.1-1.7)	0 (0-0.3)	0 (0-0.2)
Virginia	8.9	0.1 (0.1-1)	4.1 (1.6-33.4)	0 (0-0.4)	0 (0-0.3)
Washington	7.9	0.1 (0-0.8)	3 (1.2-23.1)	0 (0-0.3)	0 (0-0.3)
West Virginia	9.8	0.2 (0.1-1.3)	3.5 (1.4-21.9)	0.2 (0.1-1.2)	0.1 (0.1-0.8)
Wisconsin	9.8	0.3 (0.1-1.4)	7.1 (3-37.7)	0.1 (0.1-0.7)	0.1 (0-0.5)
Wyoming	4.7	0 (0-0.2)	0 (0-0.6)	0 (0-0.1)	0 (0-0.1)

eTable 5g: Death due to dementia associated with $PM_{2.5}$ in the contiguous US and by state

Location	$PM_{2.5}$ (μg/m^3)	PAF (%) (95% UI)	Number of deaths (95% UI)	Rate (per 100,000) (95% UI)	Age-standardized rate (per 100,000) (95% UI)

© 2019 Bowe B et al. *JAMA Network Open.*

Contiguous US	10.1	8.2 (6-13.1)	19851.5 (14420.6-31621.4)	6.1 (4.5-9.8)	5.1 (3.7-8.2)
Alabama	11.6	9.1 (6.6-14.5)	402.4 (293.2-640.4)	8.3 (6-13.1)	7.1 (5.2-11.3)
Arizona	9.5	7.8 (5.7-12.6)	345.9 (249-555.6)	4.9 (3.5-7.9)	4 (2.9-6.4)
Arkansas	10.3	8.5 (6.2-13.5)	191.7 (136.6-307.1)	6.4 (4.5-10.2)	5.2 (3.7-8.4)
California	12.2	9.2 (6.7-14.7)	2078.8 (1512.3-3299.1)	5.3 (3.8-8.3)	4.7 (3.4-7.5)
Colorado	7.3	6.4 (4.6-10.3)	230.3 (166.5-373.7)	4.1 (3-6.7)	4.3 (3.1-7)
Connecticut	8.5	7.4 (5.4-11.9)	242.8 (175.9-389.5)	6.8 (4.9-10.9)	4.5 (3.3-7.3)
Delaware	9.7	8.2 (5.9-13)	65.6 (47.5-104.9)	6.8 (4.9-10.9)	5.3 (3.8-8.5)
District of Columbia	9.7	8.2 (6-13.1)	24.1 (17.1-39.6)	3.5 (2.5-5.7)	3.4 (2.4-5.6)
Florida	7.6	6.7 (4.9-10.8)	1084.2 (776.3-1734.2)	5.2 (3.7-8.3)	3.2 (2.3-5.1)
Georgia	11.0	8.8 (6.4-14)	612.2 (444.8-978.4)	5.9 (4.3-9.4)	6.6 (4.8-10.5)
Idaho	9.2	7.8 (5.6-12.4)	100.9 (72-162.5)	5.9 (4.2-9.5)	5.5 (3.9-8.9)
Illinois	13.3	9.7 (7.1-15.5)	944.4 (684.1-1496)	7.4 (5.3-11.7)	6 (4.3-9.5)
Indiana	12.2	9.3 (6.8-14.9)	542.6 (394.2-858.1)	8.1 (5.9-12.9)	6.9 (5-10.9)
Iowa	9.3	7.9 (5.8-12.7)	253.6 (185.1-404.1)	8.1 (5.9-12.8)	5.6 (4.1-8.9)
Kansas	9.4	8 (5.8-12.7)	192.2 (138-307.2)	6.6 (4.7-10.5)	5.1 (3.7-8.2)
Kentucky	11.2	8.9 (6.5-14.2)	364.2 (264.9-582.2)	8.2 (5.9-13.1)	7.2 (5.2-11.5)
Louisiana	9.2	7.9 (5.8-12.6)	264.4 (191.1-422.6)	5.6 (4.1-9)	5.3 (3.8-8.4)
Maine	7.5	6.7 (4.9-10.9)	111.6 (79.4-178.6)	8.4 (5.9-13.4)	5.6 (4-9)
Maryland	10.3	8.5 (6.2-13.5)	346.9 (251.4-551.8)	5.7 (4.2-9.1)	4.9 (3.6-7.8)
Massachusetts	7.9	7 (5.1-11.2)	517.2 (371-827.7)	7.5 (5.4-12.1)	5.5 (4-8.8)
Michigan	10.7	8.6 (6.3-13.7)	688.6 (503.6-1099.3)	6.9 (5.1-11)	5.3 (3.9-8.5)
Minnesota	7.9	7 (5.1-11.2)	379.6 (273.3-612.1)	6.8 (4.9-11)	5.3 (3.8-8.6)
Mississippi	10.1	8.4 (6.1-13.4)	193.4 (140.3-307.5)	6.5 (4.7-10.3)	5.9 (4.3-9.4)
Missouri	10.6	8.6 (6.3-13.8)	428.8 (312.1-683.3)	7 (5.1-11.2)	5.4 (4-8.7)
Montana	7.1	6.2 (4.5-10.1)	57.7 (41.5-93.9)	5.5 (3.9-8.9)	4.2 (3-6.8)
Nebraska	8.6	7.4 (5.4-11.9)	122.5 (88.1-197.1)	6.4 (4.6-10.3)	4.9 (3.6-8)
Nevada	9.0	7.7 (5.6-12.3)	108.5 (77.7-175)	3.6 (2.6-5.8)	3.8 (2.7-6.1)
New Hampshire	7.5	6.7 (4.9-10.9)	91.9 (65.6-146.9)	6.8 (4.9-10.9)	5.1 (3.6-8.2)

© 2019 Bowe B et al. *JAMA Network Open.*

New Jersey	9.9	8.3 (6-13.2)	517.5 (373.5-822.3)	5.7 (4.1-9.1)	4.3 (3.1-6.8)
New Mexico	6.7	6 (4.3-9.7)	76.4 (54.7-123.6)	3.7 (2.6-5.9)	3 (2.2-4.9)
New York	9.4	8 (5.8-12.8)	872 (634.6-1378.7)	4.4 (3.2-6.9)	3.2 (2.4-5.1)
North Carolina	10.3	8.5 (6.2-13.5)	762.1 (551.3-1214.6)	7.4 (5.4-11.8)	6.6 (4.8-10.5)
North Dakota	6.0	5.3 (3.7-8.6)	33 (23-54.5)	4.4 (3-7.2)	3.1 (2.2-5.2)
Ohio	12.0	9.2 (6.7-14.7)	1030.2 (749.2-1641.3)	8.8 (6.4-14.1)	6.7 (4.9-10.7)
Oklahoma	9.6	8.1 (5.9-13)	247 (178.3-392.8)	6.3 (4.5-10)	5.5 (4-8.8)
Oregon	8.6	7.5 (5.5-12)	301 (219.9-484)	7.3 (5.3-11.7)	5.8 (4.3-9.4)
Pennsylvania	10.9	8.7 (6.4-13.9)	1048.9 (769.5-1663.1)	8.2 (6-13)	5.3 (3.9-8.5)
Rhode Island	7.1	6.4 (4.6-10.3)	78 (55.9-125.5)	7.4 (5.3-11.8)	4.9 (3.5-7.8)
South Carolina	10.0	8.3 (6.1-13.3)	379.2 (274.7-607.8)	7.5 (5.5-12.1)	6.7 (4.8-10.7)
South Dakota	6.6	5.8 (4.1-9.4)	36.9 (26.3-60.2)	4.2 (3-6.9)	3.1 (2.2-5)
Tennessee	10.2	8.4 (6.2-13.5)	537.8 (388.4-851.1)	8 (5.8-12.7)	7.1 (5.1-11.2)
Texas	10.3	8.5 (6.2-13.5)	1310.5 (959.6-2087.8)	4.6 (3.4-7.4)	5.3 (3.9-8.4)
Utah	7.6	6.7 (4.9-10.9)	120.2 (85.7-192.6)	3.9 (2.8-6.2)	5.1 (3.6-8.2)
Vermont	6.9	6.2 (4.4-10)	40 (28.4-63.9)	6.4 (4.6-10.2)	4.6 (3.3-7.4)
Virginia	8.9	7.7 (5.6-12.4)	496.7 (361.9-789.7)	5.9 (4.3-9.3)	5.4 (3.9-8.5)
Washington	7.9	7 (5.1-11.2)	385 (277-618.6)	5.2 (3.7-8.4)	4.8 (3.4-7.7)
West Virginia	9.8	8.2 (6-13.1)	140.9 (101.4-225.3)	7.8 (5.6-12.4)	5.6 (4-8.9)
Wisconsin	9.8	8.1 (5.9-13)	438.3 (318.7-689.9)	7.6 (5.5-11.9)	5.7 (4.1-9)
Wyoming	4.7	3.8 (2.6-6.3)	13 (8.8-22)	2.3 (1.5-3.8)	2 (1.4-3.4)
Abbreviations: $PM_{2.5}$, ambient fine particulate matter; PAF, population attributable fraction; UI, uncertainty interval					

eTable 5h: Death due to type 2 diabetes associated with $PM_{2.5}$ in the contiguous US and by state

© 2019 Bowe B et al. *JAMA Network Open.*

Location	$PM_{2.5}$ (μg/m^3)	PAF (%) (95% UI)	Number of deaths (95% UI)	Rate (per 100,000) (95% UI)	Age-standardized rate (per 100,000) (95% UI)
Contiguous US	10.1	1.4 (1.2-1.5)	501.3 (447.5-561.1)	0.2 (0.1-0.2)	0.1 (0.1-0.2)
Alabama	11.6	1.6 (1.4-1.8)	5.2 (4.4-6.1)	0.1 (0.1-0.1)	0.1 (0.1-0.1)
Arizona	9.5	0.6 (0.5-0.7)	4.8 (4.2-5.6)	0.1 (0.1-0.1)	0.1 (0-0.1)
Arkansas	10.3	0.7 (0.6-0.8)	1.7 (1.5-2.1)	0.1 (0-0.1)	0 (0-0.1)
California	12.2	4.8 (4.3-5.4)	299.9 (269-333.1)	0.8 (0.7-0.8)	0.7 (0.6-0.8)
Colorado	7.3	0.1 (0.1-0.2)	0.6 (0.5-0.7)	0 (0-0)	0 (0-0)
Connecticut	8.5	0.3 (0.2-0.3)	0.4 (0.3-0.5)	0 (0-0)	0 (0-0)
Delaware	9.7	0.5 (0.4-0.5)	0.4 (0.3-0.5)	0 (0-0.1)	0 (0-0)
District of Columbia	9.7	0.5 (0.4-0.5)	0.1 (0.1-0.2)	0 (0-0)	0 (0-0)
Florida	7.6	0.2 (0.1-0.2)	3.2 (2.8-3.6)	0 (0-0)	0 (0-0)
Georgia	11.0	1.1 (0.9-1.2)	5.3 (4.5-6.1)	0.1 (0-0.1)	0 (0-0.1)
Idaho	9.2	0.6 (0.5-0.6)	0.9 (0.7-1.1)	0.1 (0-0.1)	0 (0-0.1)
Illinois	13.3	5 (4.4-5.5)	40.6 (35.7-45.9)	0.3 (0.3-0.4)	0.3 (0.2-0.3)
Indiana	12.2	2.3 (2.1-2.6)	18 (15.7-20.8)	0.3 (0.2-0.3)	0.2 (0.2-0.3)
Iowa	9.3	0.4 (0.4-0.4)	1 (0.9-1.2)	0 (0-0)	0 (0-0)
Kansas	9.4	0.4 (0.4-0.5)	1.3 (1.1-1.5)	0 (0-0.1)	0 (0-0)
Kentucky	11.2	1.4 (1.2-1.6)	6.7 (5.7-7.7)	0.1 (0.1-0.2)	0.1 (0.1-0.1)
Louisiana	9.2	0.4 (0.3-0.4)	0.8 (0.6-0.9)	0 (0-0)	0 (0-0)
Maine	7.5	0.1 (0.1-0.1)	0.2 (0.1-0.2)	0 (0-0)	0 (0-0)
Maryland	10.3	0.7 (0.6-0.8)	2.8 (2.4-3.2)	0 (0-0.1)	0 (0-0)
Massachusetts	7.9	0.2 (0.1-0.2)	0.5 (0.4-0.6)	0 (0-0)	0 (0-0)
Michigan	10.7	1 (0.9-1.1)	9.1 (7.9-10.4)	0.1 (0.1-0.1)	0.1 (0.1-0.1)
Minnesota	7.9	0.2 (0.2-0.2)	1 (0.9-1.2)	0 (0-0)	0 (0-0)
Mississippi	10.1	0.6 (0.5-0.7)	0.9 (0.7-1.1)	0 (0-0)	0 (0-0)
Missouri	10.6	0.9 (0.8-1.1)	4.1 (3.5-4.7)	0.1 (0.1-0.1)	0.1 (0-0.1)
Montana	7.1	0.1 (0.1-0.1)	0.1 (0.1-0.2)	0 (0-0)	0 (0-0)

© 2019 Bowe B et al. *JAMA Network Open.*

Nebraska	8.6	0.3 (0.3-0.3)	0.5 (0.4-0.6)	0 (0-0)	0 (0-0)
Nevada	9.0	0.3 (0.3-0.4)	0.5 (0.4-0.6)	0 (0-0)	0 (0-0)
New Hampshire	7.5	0.1 (0.1-0.1)	0.1 (0.1-0.2)	0 (0-0)	0 (0-0)
New Jersey	9.9	0.6 (0.5-0.7)	2 (1.7-2.4)	0 (0-0)	0 (0-0)
New Mexico	6.7	0.1 (0.1-0.1)	0.2 (0.2-0.2)	0 (0-0)	0 (0-0)
New York	9.4	0.5 (0.4-0.5)	3.8 (3.3-4.3)	0 (0-0)	0 (0-0)
North Carolina	10.3	0.7 (0.6-0.8)	7.1 (6.2-8.1)	0.1 (0.1-0.1)	0.1 (0.1-0.1)
North Dakota	6.0	0 (0-0.1)	0 (0-0)	0 (0-0)	0 (0-0)
Ohio	12.0	1.9 (1.7-2.2)	27.2 (23.9-30.7)	0.2 (0.2-0.3)	0.2 (0.2-0.2)
Oklahoma	9.6	0.5 (0.4-0.5)	2.1 (1.8-2.4)	0.1 (0-0.1)	0 (0-0.1)
Oregon	8.6	0.3 (0.2-0.3)	1.4 (1.2-1.7)	0 (0-0)	0 (0-0)
Pennsylvania	10.9	1.2 (1-1.3)	13.9 (12.2-15.8)	0.1 (0.1-0.1)	0.1 (0.1-0.1)
Rhode Island	7.1	0.1 (0.1-0.1)	0.1 (0-0.1)	0 (0-0)	0 (0-0)
South Carolina	10.0	0.6 (0.5-0.7)	2.2 (1.9-2.5)	0 (0-0.1)	0 (0-0)
South Dakota	6.6	0.1 (0.1-0.1)	0.1 (0.1-0.1)	0 (0-0)	0 (0-0)
Tennessee	10.2	0.7 (0.6-0.8)	4.6 (4-5.3)	0.1 (0.1-0.1)	0.1 (0-0.1)
Texas	10.3	0.8 (0.7-0.9)	15.9 (13.9-18.1)	0.1 (0-0.1)	0.1 (0.1-0.1)
Utah	7.6	0.2 (0.1-0.2)	0.3 (0.3-0.4)	0 (0-0)	0 (0-0)
Vermont	6.9	0.1 (0.1-0.1)	0.1 (0-0.1)	0 (0-0)	0 (0-0)
Virginia	8.9	0.3 (0.3-0.3)	1.9 (1.6-2.2)	0 (0-0)	0 (0-0)
Washington	7.9	0.2 (0.2-0.3)	1.8 (1.5-2)	0 (0-0)	0 (0-0)
West Virginia	9.8	0.5 (0.5-0.6)	1.8 (1.5-2.1)	0.1 (0.1-0.1)	0.1 (0.1-0.1)
Wisconsin	9.8	0.7 (0.6-0.7)	4.2 (3.7-4.8)	0.1 (0.1-0.1)	0.1 (0-0.1)
Wyoming	4.7	0 (0-0)	0 (0-0)	0 (0-0)	0 (0-0)
Abbreviations: $PM_{2.5}$, ambient fine particulate matter; PAF, population attributable fraction; UI, uncertainty interval					

© 2019 Bowe B et al. *JAMA Network Open.*

eTable 5i: Death due to hypertension associated with $PM_{2.5}$ in the contiguous US and by state

Location	$PM_{2.5}$ (μg/m^3)	PAF (%) (95% UI)	Number of deaths (95% UI)	Rate (per 100,000) (95% UI)	Age-standardized rate (per 100,000) (95% UI)
Contiguous US	10.1	34.1 (30.6-37.6)	30696.9 (27518.1-33881.9)	9.5 (8.5-10.5)	8 (7.2-8.8)
Alabama	11.6	37 (33.3-40.8)	507.3 (448.7-563.9)	10.4 (9.2-11.6)	8.7 (7.8-9.7)
Arizona	9.5	32.8 (29.3-36.2)	753.7 (668.2-838.5)	10.7 (9.5-12)	8.7 (7.7-9.7)
Arkansas	10.3	35 (31.4-38.6)	449 (391.5-503.1)	14.9 (13-16.7)	12.4 (10.8-13.9)
California	12.2	37.4 (33.6-41.2)	4346.9 (3882.7-4790.4)	11 (9.8-12.1)	9.9 (8.8-10.9)
Colorado	7.3	27.6 (24.6-30.6)	278.4 (242.4-314.8)	5 (4.3-5.6)	4.7 (4.1-5.4)
Connecticut	8.5	31.3 (28-34.7)	223.5 (194.7-255.3)	6.2 (5.4-7.1)	4.4 (3.9-5.1)
Delaware	9.7	33.9 (30.4-37.5)	71.8 (58.7-84.1)	7.5 (6.1-8.7)	5.9 (4.8-6.9)
District of Columbia	9.7	34 (30.4-37.5)	118.6 (100.2-137.7)	17.1 (14.4-19.8)	17.5 (14.8-20.3)
Florida	7.6	28.8 (25.7-32)	1862.5 (1649.6-2071.2)	8.9 (7.9-9.9)	6 (5.3-6.7)
Georgia	11.0	36.1 (32.4-39.8)	1224.4 (1094.4-1356.3)	11.7 (10.5-13)	11.5 (10.2-12.7)
Idaho	9.2	32.5 (29.1-35.9)	97 (81.5-112.4)	5.7 (4.7-6.5)	5.2 (4.3-6)
Illinois	13.3	39.2 (35.3-43.1)	1326.4 (1178.9-1462.3)	10.4 (9.2-11.4)	8.5 (7.6-9.4)
Indiana	12.2	38 (34.1-41.8)	653 (579.4-726.8)	9.8 (8.7-10.9)	8.3 (7.4-9.3)
Iowa	9.3	33.1 (29.7-36.6)	310.4 (270.8-347.1)	9.9 (8.6-11)	7.1 (6.2-7.9)
Kansas	9.4	33.2 (29.7-36.7)	214.8 (187.1-244.3)	7.4 (6.4-8.4)	5.8 (5-6.6)
Kentucky	11.2	36.5 (32.8-40.2)	456.6 (400.2-511.6)	10.3 (9-11.5)	8.6 (7.6-9.7)
Louisiana	9.2	33 (29.6-36.5)	498.3 (439.2-559.5)	10.6 (9.4-11.9)	9.5 (8.3-10.7)
Maine	7.5	28.9 (25.8-32.1)	75.4 (63.3-87.9)	5.6 (4.7-6.6)	3.9 (3.2-4.6)
Maryland	10.3	35 (31.4-38.6)	690.1 (616.4-765.5)	11.4 (10.2-12.6)	9.6 (8.6-10.7)
Massachusetts	7.9	29.8 (26.6-33)	371.7 (324.5-416.4)	5.4 (4.7-6.1)	4.1 (3.6-4.6)
Michigan	10.7	35.4 (31.8-39.1)	1105.2 (981.2-1217.3)	11.1 (9.8-12.2)	8.6 (7.7-9.5)
Minnesota	7.9	29.8 (26.6-33)	315.6 (276-356.3)	5.7 (4.9-6.4)	4.4 (3.8-5)
Mississippi	10.1	34.7 (31.1-38.3)	571 (506.8-633.7)	19.1 (17-21.2)	16.6 (14.7-18.4)
Missouri	10.6	35.5 (31.9-39.2)	584.2 (517.8-651.5)	9.6 (8.5-10.7)	7.6 (6.7-8.4)

© 2019 Bowe B et al. *JAMA Network Open.*

Montana	7.1	27.1 (24.1-30.1)	59.7 (49.2-70.3)	5.7 (4.7-6.7)	4.3 (3.5-5)
Nebraska	8.6	31.4 (28.1-34.7)	149.4 (127.4-170.9)	7.8 (6.6-8.9)	6.1 (5.1-6.9)
Nevada	9.0	32.3 (28.9-35.7)	348.6 (307.1-390.3)	11.6 (10.2-13)	10.9 (9.6-12.2)
New Hampshire	7.5	28.9 (25.8-32.1)	77.7 (65.1-91.2)	5.8 (4.8-6.8)	4.2 (3.5-5)
New Jersey	9.9	34.2 (30.7-37.8)	613.7 (541.4-686.4)	6.8 (6-7.6)	5.3 (4.7-6)
New Mexico	6.7	26.2 (23.4-29.2)	107.9 (91.5-124.7)	5.2 (4.4-6)	4.2 (3.6-4.9)
New York	9.4	33.3 (29.8-36.8)	2267.9 (2019.8-2514.1)	11.4 (10.2-12.7)	8.9 (7.9-9.8)
North Carolina	10.3	34.9 (31.3-38.6)	789.8 (698.4-881.7)	7.7 (6.8-8.6)	6.6 (5.8-7.4)
North Dakota	6.0	23.5 (20.9-26.2)	38.7 (31.2-46.5)	5.1 (4.1-6.2)	4 (3.2-4.9)
Ohio	12.0	37.6 (33.8-41.4)	1289.6 (1150.8-1433)	11.1 (9.9-12.3)	8.6 (7.7-9.6)
Oklahoma	9.6	33.8 (30.3-37.3)	1067.4 (952-1186.9)	27.2 (24.2-30.2)	23.5 (20.9-26.2)
Oregon	8.6	31.6 (28.3-35)	290.6 (254-325.1)	7 (6.1-7.8)	5.6 (4.8-6.2)
Pennsylvania	10.9	35.8 (32.2-39.5)	1009.9 (896.5-1118.3)	7.9 (7-8.7)	5.6 (5-6.2)
Rhode Island	7.1	27.7 (24.7-30.8)	79.8 (67-93)	7.5 (6.3-8.8)	5.4 (4.5-6.3)
South Carolina	10.0	34.5 (31-38.1)	444.2 (392.5-497.9)	8.8 (7.8-9.9)	7.4 (6.6-8.3)
South Dakota	6.6	25.4 (22.6-28.3)	71.1 (59.6-82.9)	8.2 (6.9-9.5)	6.1 (5.2-7.2)
Tennessee	10.2	34.9 (31.3-38.5)	778.3 (687.3-861.7)	11.6 (10.2-12.8)	10 (8.8-11.1)
Texas	10.3	35 (31.4-38.6)	2236.2 (2006-2479.1)	7.9 (7.1-8.8)	8.3 (7.4-9.2)
Utah	7.6	28.9 (25.8-32)	105.7 (89.5-122)	3.4 (2.9-3.9)	4.2 (3.6-4.9)
Vermont	6.9	26.8 (23.8-29.7)	60.4 (49-71.9)	9.7 (7.9-11.5)	7 (5.6-8.3)
Virginia	8.9	32.4 (29-35.9)	502.4 (442.3-564.2)	5.9 (5.2-6.7)	5.3 (4.6-5.9)
Washington	7.9	29.7 (26.6-33)	544.1 (478.1-611)	7.3 (6.5-8.2)	6.6 (5.8-7.4)
West Virginia	9.8	34 (30.5-37.6)	188.5 (162.9-214.3)	10.4 (9-11.8)	7.9 (6.8-8.9)
Wisconsin	9.8	33.8 (30.3-37.3)	452.1 (397.8-506.4)	7.8 (6.9-8.7)	6 (5.3-6.7)
Wyoming	4.7	17.5 (15.5-19.6)	17.4 (13.3-21.5)	3 (2.3-3.7)	2.5 (1.9-3.1)
Abbreviations: $PM_{2.5}$, ambient fine particulate matter; PAF, population attributable fraction; UI, uncertainty interval					

© 2019 Bowe B et al. *JAMA Network Open.*

eTable 5j: Death due to lung cancer associated with $PM_{2.5}$ in the contiguous US and by state

Location	$PM_{2.5}$ (μg/m^3)	PAF (%) (95% UI)	Number of deaths (95% UI)	Rate (per 100,000) (95% UI)	Age-standardized rate (per 100,000) (95% UI)
Contiguous US	10.1	12.1 (10.4-14.1)	17545.3 (15055.3-20464.5)	5.4 (4.7-6.3)	4.4 (3.8-5.1)
Alabama	11.6	13.3 (11.4-15.5)	392.2 (334.1-457.2)	8 (6.9-9.4)	6.2 (5.3-7.2)
Arizona	9.5	11.5 (9.9-13.5)	319.9 (271.6-374.5)	4.6 (3.9-5.3)	3.5 (3-4.1)
Arkansas	10.3	12.4 (10.7-14.5)	243.8 (208-287.9)	8.1 (6.9-9.6)	6.3 (5.4-7.5)
California	12.2	13.5 (11.6-15.7)	1554.9 (1327.7-1807.1)	3.9 (3.4-4.6)	3.6 (3.1-4.2)
Colorado	7.3	9.4 (8.1-11.1)	141.4 (120.2-166.5)	2.5 (2.1-3)	2.3 (2-2.8)
Connecticut	8.5	10.9 (9.4-12.8)	164.5 (139.3-194.4)	4.6 (3.9-5.4)	3.4 (2.9-4.1)
Delaware	9.7	12 (10.3-14)	66 (55.6-78.6)	6.9 (5.8-8.2)	5 (4.2-5.9)
District of Columbia	9.7	12 (10.3-14)	24.4 (19.6-30.1)	3.5 (2.8-4.3)	3.7 (3-4.6)
Florida	7.6	9.9 (8.5-11.6)	1131.9 (963.5-1325.3)	5.4 (4.6-6.3)	3.6 (3-4.2)
Georgia	11.0	12.9 (11.1-15.1)	568.4 (484.9-667.4)	5.4 (4.6-6.4)	5 (4.3-5.9)
Idaho	9.2	11.4 (9.8-13.3)	69.4 (58-82.8)	4 (3.4-4.8)	3.4 (2.9-4.1)
Illinois	13.3	14.2 (12.2-16.6)	855.1 (731.6-994.1)	6.7 (5.7-7.8)	5.6 (4.8-6.5)
Indiana	12.2	13.7 (11.8-16)	519.4 (443.7-604.8)	7.8 (6.7-9.1)	6.5 (5.5-7.5)
Iowa	9.3	11.7 (10-13.6)	188.9 (159.5-221.5)	6 (5.1-7)	4.6 (3.9-5.4)
Kansas	9.4	11.7 (10-13.7)	162.7 (138.1-193)	5.6 (4.7-6.6)	4.6 (3.9-5.5)
Kentucky	11.2	13.1 (11.2-15.2)	412.1 (348.6-480.1)	9.3 (7.8-10.8)	7.4 (6.3-8.6)
Louisiana	9.2	11.6 (10-13.6)	297.4 (252.3-348)	6.3 (5.4-7.4)	5.4 (4.6-6.3)
Maine	7.5	10 (8.5-11.6)	91.5 (77.2-109)	6.9 (5.8-8.2)	4.5 (3.8-5.3)
Maryland	10.3	12.4 (10.7-14.5)	320.6 (273.6-374.8)	5.3 (4.5-6.2)	4.5 (3.8-5.2)
Massachusetts	7.9	10.3 (8.8-12)	316.4 (270-372.5)	4.6 (3.9-5.4)	3.6 (3.1-4.3)
Michigan	10.7	12.6 (10.8-14.7)	696.1 (596.7-812.8)	7 (6-8.2)	5.4 (4.6-6.3)
Minnesota	7.9	10.3 (8.8-12.1)	238.5 (202.8-277.2)	4.3 (3.6-5)	3.5 (3-4.1)
Mississippi	10.1	12.3 (10.6-14.4)	222.1 (189-263.7)	7.4 (6.3-8.8)	6.1 (5.2-7.2)
Missouri	10.6	12.7 (10.9-14.8)	467.6 (398.1-549.5)	7.6 (6.5-9)	5.9 (5-6.9)

© 2019 Bowe B et al. *JAMA Network Open.*

Montana	7.1	9.2 (7.9-10.8)	43 (35.6-51.9)	4.1 (3.4-4.9)	3 (2.5-3.6)
Nebraska	8.6	11 (9.4-12.8)	89.7 (75.4-104.8)	4.7 (3.9-5.5)	3.9 (3.3-4.6)
Nevada	9.0	11.3 (9.7-13.2)	148.8 (126.8-175.9)	5 (4.2-5.9)	4.3 (3.7-5.1)
New Hampshire	7.5	10 (8.5-11.6)	69.8 (58.9-82.7)	5.2 (4.4-6.2)	3.8 (3.2-4.6)
New Jersey	9.9	12.1 (10.4-14.2)	432.7 (366.9-505.5)	4.8 (4.1-5.6)	3.8 (3.2-4.5)
New Mexico	6.7	8.9 (7.6-10.4)	60.3 (50.3-71.5)	2.9 (2.4-3.4)	2.3 (1.9-2.7)
New York	9.4	11.7 (10.1-13.7)	918.7 (785.4-1075.7)	4.6 (4-5.4)	3.7 (3.1-4.3)
North Carolina	10.3	12.4 (10.7-14.5)	657.3 (556.1-766)	6.4 (5.4-7.5)	5.2 (4.4-6.1)
North Dakota	6.0	7.8 (6.7-9.2)	24.2 (20.1-29.2)	3.2 (2.7-3.9)	2.7 (2.3-3.3)
Ohio	12.0	13.5 (11.6-15.8)	922.3 (790.5-1078.7)	7.9 (6.8-9.3)	6.1 (5.2-7.1)
Oklahoma	9.6	11.9 (10.2-13.9)	264.1 (225-308.7)	6.7 (5.7-7.9)	5.6 (4.8-6.5)
Oregon	8.6	11 (9.5-12.9)	205.8 (174.7-242.4)	5 (4.2-5.9)	3.9 (3.3-4.5)
Pennsylvania	10.9	12.8 (11-14.9)	885.3 (757.5-1029.7)	6.9 (5.9-8)	5 (4.2-5.8)
Rhode Island	7.1	9.5 (8.1-11.1)	52.6 (44.1-62.8)	5 (4.2-5.9)	3.7 (3.1-4.4)
South Carolina	10.0	12.3 (10.5-14.3)	331.2 (283-390.6)	6.6 (5.6-7.8)	5 (4.3-6)
South Dakota	6.6	8.6 (7.3-10)	36.2 (29.8-43.2)	4.2 (3.4-5)	3.3 (2.7-3.9)
Tennessee	10.2	12.4 (10.6-14.5)	496.6 (423.5-579)	7.4 (6.3-8.6)	5.9 (5-6.9)
Texas	10.3	12.4 (10.7-14.5)	1133 (965.9-1321.8)	4 (3.4-4.7)	4.1 (3.5-4.7)
Utah	7.6	10 (8.5-11.6)	44.8 (36.6-54)	1.4 (1.2-1.7)	1.7 (1.4-2.1)
Vermont	6.9	9.1 (7.8-10.6)	32.4 (26.8-38.7)	5.2 (4.3-6.2)	3.6 (3-4.4)
Virginia	8.9	11.4 (9.7-13.3)	421.9 (359.7-493.8)	5 (4.2-5.8)	4.2 (3.6-4.9)
Washington	7.9	10.3 (8.8-12)	301.8 (255.8-355.2)	4.1 (3.5-4.8)	3.5 (3-4.1)
West Virginia	9.8	12 (10.3-14.1)	161.2 (136.4-189.3)	8.9 (7.5-10.4)	6 (5.1-7)
Wisconsin	9.8	11.9 (10.2-13.9)	334.3 (283.7-389)	5.8 (4.9-6.7)	4.5 (3.8-5.2)
Wyoming	4.7	5.6 (4.8-6.6)	12.1 (9.5-15)	2.1 (1.6-2.6)	1.7 (1.3-2.1)
Abbreviations: $PM_{2.5}$, ambient fine particulate matter; PAF, population attributable fraction; UI, uncertainty interval					

© 2019 Bowe B et al. *JAMA Network Open.*

eTable 5k: Death due to pneumonia associated with $PM_{2.5}$ in the contiguous US and by state

Location	$PM_{2.5}$ (μg/m^3)	PAF (%) (95% UI)	Number of deaths (95% UI)	Rate (per 100,000) (95% UI)	Age-standardized rate (per 100,000) (95% UI)
Contiguous US	10.1	18 (15.7-21.9)	8854.9 (7696.2-10710.6)	2.7 (2.4-3.3)	2.3 (2-2.8)
Alabama	11.6	21.8 (19.2-25.9)	243.2 (211.3-289.3)	5 (4.3-5.9)	4.2 (3.6-4.9)
Arizona	9.5	16.6 (14.3-20.4)	125.6 (107-152.9)	1.8 (1.5-2.2)	1.4 (1.2-1.8)
Arkansas	10.3	18.8 (16.4-22.7)	121.5 (104.4-146.7)	4 (3.5-4.9)	3.3 (2.9-4)
California	12.2	22.7 (20.1-26.8)	1291.1 (1143.6-1518.4)	3.3 (2.9-3.8)	3 (2.6-3.5)
Colorado	7.3	10.9 (8.8-14.3)	48.1 (38.2-63.5)	0.9 (0.7-1.1)	0.8 (0.7-1.1)
Connecticut	8.5	14.2 (11.8-17.9)	85.7 (69.6-107.4)	2.4 (1.9-3)	1.7 (1.3-2.1)
Delaware	9.7	17.4 (14.9-21.3)	28.4 (22.6-35.9)	3 (2.3-3.7)	2.3 (1.8-2.9)
District of Columbia	9.7	17.4 (14.9-21.3)	12.3 (9-16.3)	1.8 (1.3-2.3)	1.8 (1.3-2.3)
Florida	7.6	11.6 (9.4-15.1)	324.2 (259.8-431.3)	1.5 (1.2-2.1)	1 (0.8-1.4)
Georgia	11.0	20.4 (18-24.4)	270.4 (234.4-323.4)	2.6 (2.2-3.1)	2.7 (2.3-3.2)
Idaho	9.2	16 (13.7-19.9)	30.2 (24.2-38)	1.8 (1.4-2.2)	1.6 (1.3-2.1)
Illinois	13.3	25.3 (22.6-29.6)	547.1 (488.6-638.9)	4.3 (3.8-5)	3.6 (3.2-4.2)
Indiana	12.2	23.2 (20.6-27.3)	216.5 (187-255.6)	3.2 (2.8-3.8)	2.8 (2.4-3.3)
Iowa	9.3	16.3 (13.9-20.1)	74.5 (61.2-91.9)	2.4 (1.9-2.9)	1.7 (1.4-2.1)
Kansas	9.4	16.5 (14.1-20.4)	71.3 (59.3-88.8)	2.4 (2-3)	1.9 (1.6-2.4)
Kentucky	11.2	21 (18.5-25)	172 (148.1-204.2)	3.9 (3.3-4.6)	3.3 (2.9-4)
Louisiana	9.2	16.2 (13.8-20)	114.8 (94.5-141.2)	2.5 (2-3)	2.2 (1.8-2.7)
Maine	7.5	11.6 (9.3-15.1)	26.9 (20.4-36.5)	2 (1.5-2.7)	1.4 (1-1.9)
Maryland	10.3	18.8 (16.4-22.7)	168.4 (143.4-203.6)	2.8 (2.4-3.4)	2.4 (2-2.9)
Massachusetts	7.9	12.4 (10.1-16)	157.9 (127.2-205)	2.3 (1.9-3)	1.8 (1.4-2.3)
Michigan	10.7	19.6 (17.2-23.5)	308.7 (268.2-374.4)	3.1 (2.7-3.8)	2.4 (2.1-3)
Minnesota	7.9	12.5 (10.3-16.1)	63.7 (50.7-82.8)	1.1 (0.9-1.5)	0.9 (0.7-1.2)
Mississippi	10.1	18.3 (15.9-22.2)	134.1 (114.6-164)	4.5 (3.8-5.5)	3.9 (3.4-4.8)
Missouri	10.6	19.6 (17.1-23.5)	212 (183.6-256.7)	3.5 (3-4.2)	2.8 (2.4-3.4)

© 2019 Bowe B et al. *JAMA Network Open.*

Montana	7.1	10.2 (8.2-13.6)	13.5 (9.8-18.9)	1.3 (0.9-1.8)	1 (0.7-1.4)
Nebraska	8.6	14.6 (12.2-18.2)	47.5 (38.3-59.6)	2.5 (2-3.1)	1.9 (1.5-2.4)
Nevada	9.0	15.5 (13.1-19.2)	93.7 (77.7-116.3)	3.1 (2.6-3.9)	2.9 (2.4-3.6)
New Hampshire	7.5	11.6 (9.3-15)	21.7 (16.5-28.6)	1.6 (1.2-2.1)	1.2 (0.9-1.6)
New Jersey	9.9	17.8 (15.4-21.7)	223.4 (189.7-270.4)	2.5 (2.1-3)	1.9 (1.7-2.4)
New Mexico	6.7	9.2 (7.2-12.5)	28.1 (21-38.3)	1.3 (1-1.8)	1.1 (0.8-1.5)
New York	9.4	16.7 (14.3-20.5)	711.2 (602.8-877)	3.6 (3-4.4)	2.8 (2.3-3.4)
North Carolina	10.3	18.8 (16.4-22.7)	339.6 (293.9-404.5)	3.3 (2.9-3.9)	2.9 (2.5-3.4)
North Dakota	6.0	7.1 (5.4-10.3)	8.9 (6.2-13.2)	1.2 (0.8-1.7)	0.9 (0.6-1.4)
Ohio	12.0	22.7 (20.1-26.8)	449.3 (392.2-529.2)	3.9 (3.4-4.5)	3 (2.6-3.5)
Oklahoma	9.6	17.2 (14.7-21.1)	89.9 (74.9-109.6)	2.3 (1.9-2.8)	2 (1.7-2.4)
Oregon	8.6	14.5 (12.1-18.2)	53.6 (42.9-69.5)	1.3 (1-1.7)	1 (0.8-1.4)
Pennsylvania	10.9	20.1 (17.7-24)	493.7 (432.5-598.5)	3.9 (3.4-4.7)	2.7 (2.3-3.2)
Rhode Island	7.1	10.4 (8.2-13.8)	17.6 (13.1-24)	1.7 (1.2-2.3)	1.2 (0.9-1.6)
South Carolina	10.0	18.2 (15.7-22)	111.5 (94.3-134.8)	2.2 (1.9-2.7)	1.9 (1.6-2.3)
South Dakota	6.6	8.9 (7-12.2)	16.1 (12-23.1)	1.9 (1.4-2.7)	1.4 (1.1-2)
Tennessee	10.2	18.7 (16.3-22.6)	283.7 (243.5-345.9)	4.2 (3.6-5.2)	3.6 (3.1-4.4)
Texas	10.3	18.9 (16.4-22.8)	480.8 (417-580.3)	1.7 (1.5-2.1)	1.8 (1.6-2.2)
Utah	7.6	11.9 (9.7-15.3)	33.5 (26.1-43.4)	1.1 (0.8-1.4)	1.4 (1-1.8)
Vermont	6.9	9.7 (7.7-13.1)	6.8 (4.7-9.7)	1.1 (0.8-1.6)	0.8 (0.5-1.1)
Virginia	8.9	15.4 (13-19.2)	171.4 (142.3-213.3)	2 (1.7-2.5)	1.8 (1.5-2.2)
Washington	7.9	12.6 (10.4-16.2)	91.5 (73.1-120.2)	1.2 (1-1.6)	1.1 (0.9-1.4)
West Virginia	9.8	17.5 (15.1-21.4)	73.3 (60.7-89.9)	4 (3.3-5)	2.9 (2.4-3.6)
Wisconsin	9.8	17.4 (15-21.2)	142.1 (119.2-171.6)	2.5 (2.1-3)	1.9 (1.6-2.3)
Wyoming	4.7	4.1 (2.9-6.6)	4.2 (2.7-7)	0.7 (0.5-1.2)	0.6 (0.4-1.1)
Abbreviations: $PM_{2.5}$, ambient fine particulate matter; PAF, population attributable fraction; UI, uncertainty interval					

© 2019 Bowe B et al. *JAMA Network Open.*

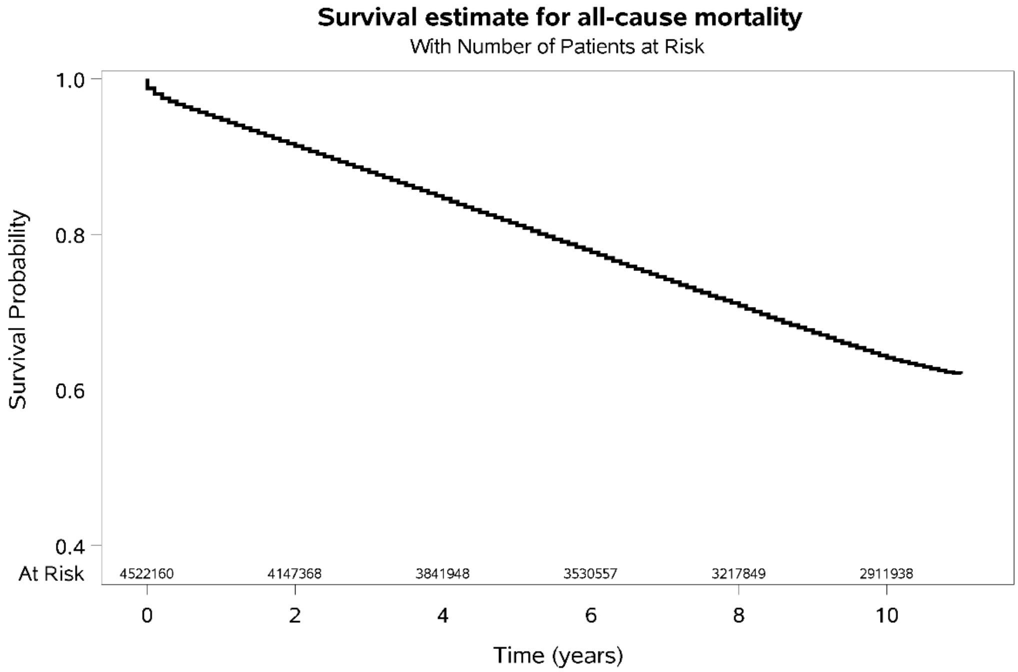

eFigure 1: Kaplan-Meier Curve of all-cause mortality. Number at risk in the cohort during follow-up is included at select time points.

© 2019 Bowe B et al. *JAMA Network Open.*

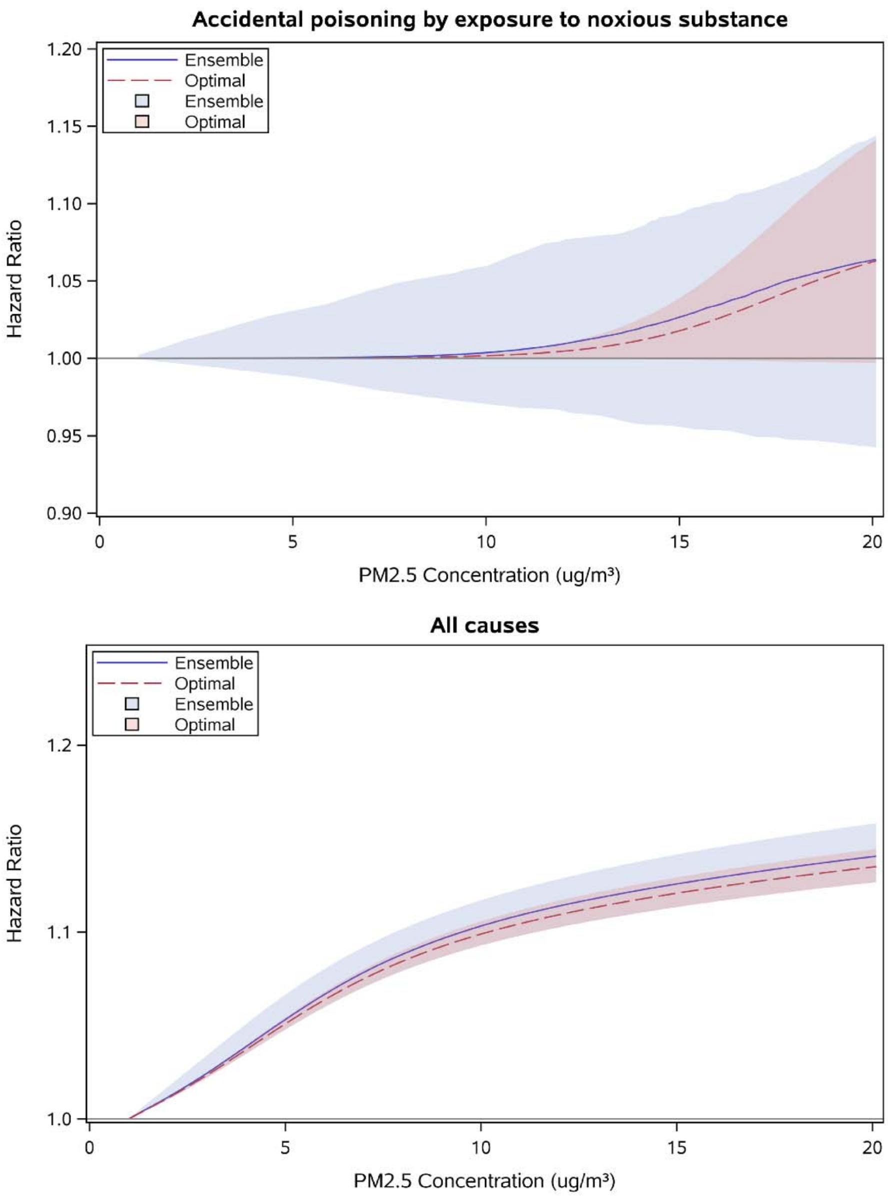

eFigure 2: Non-linear exposure-response hazard functions for negative and positive outcome controls. Curves are presented for accidental poisoning by exposure to noxious substance (negative outcome control) and all-cause (positive outcome control). Plots are presented for both the optimal and ensembled model. 95% UI are presented as bands.

© 2019 Bowe B et al. *JAMA Network Open.*

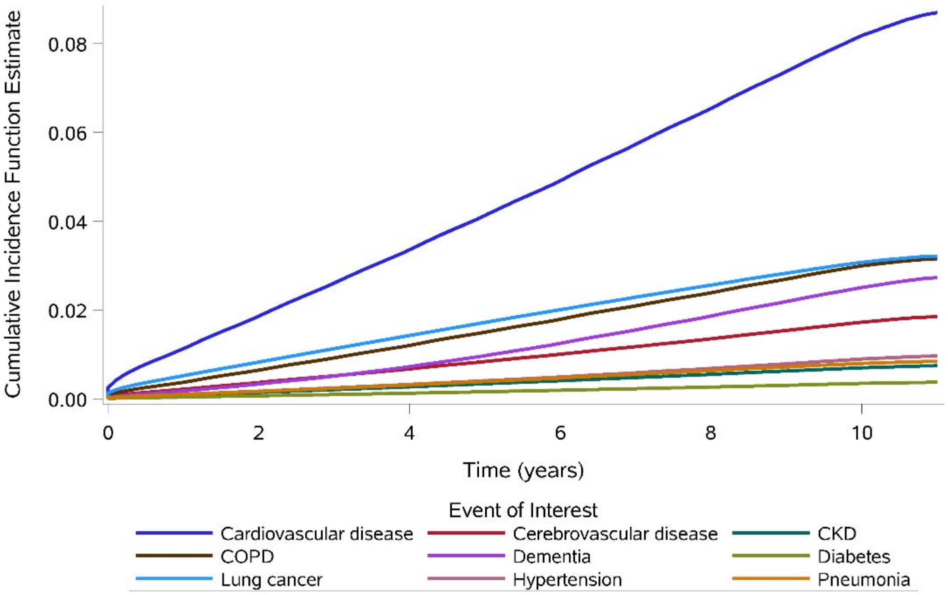

eFigure 3: Cumulative incidence of specific causes of death. Estimates of the Cumulative incidence function (%) are colored by cause of death.

© 2019 Bowe B et al. *JAMA Network Open.*

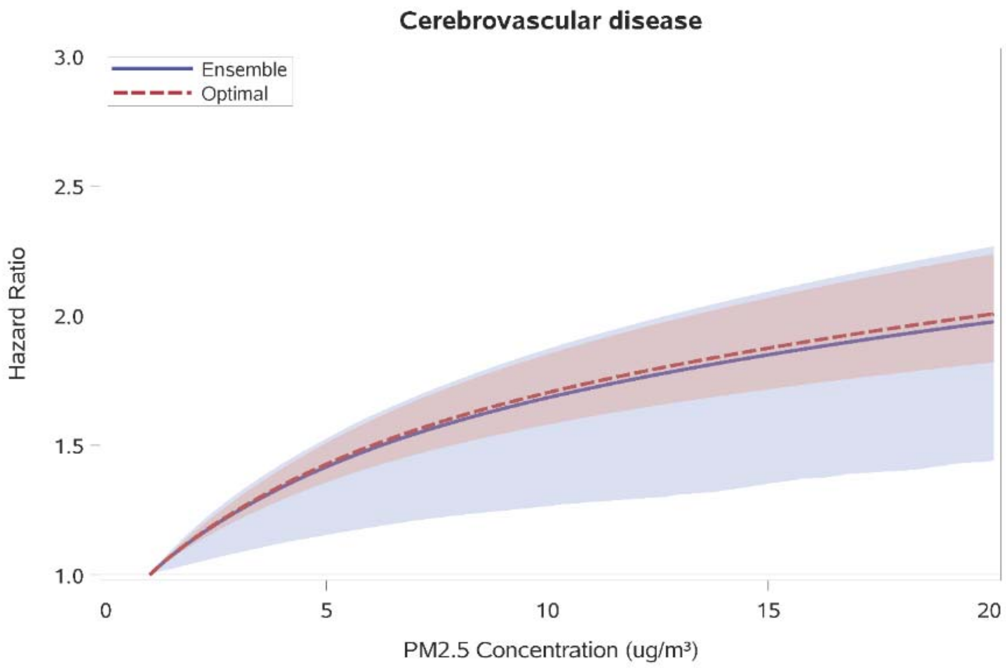

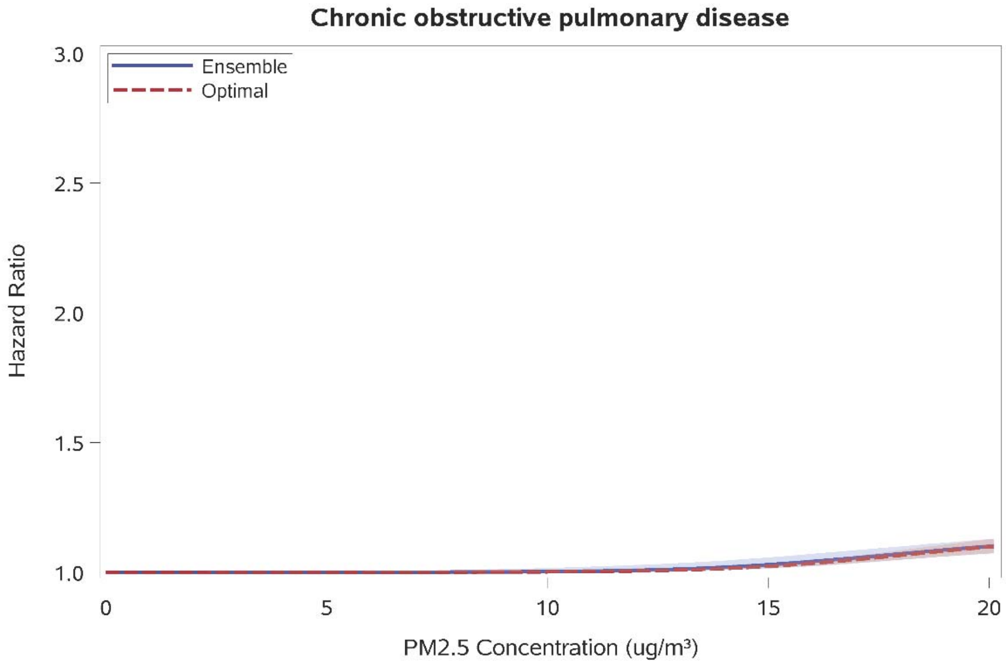

eFigure 4a: Non-linear exposure-response hazard functions for cause-specific mortality (continued). Plots are presented for both the optimal and ensembled model. 95% UI are presented as bands.

© 2019 Bowe B et al. *JAMA Network Open.*

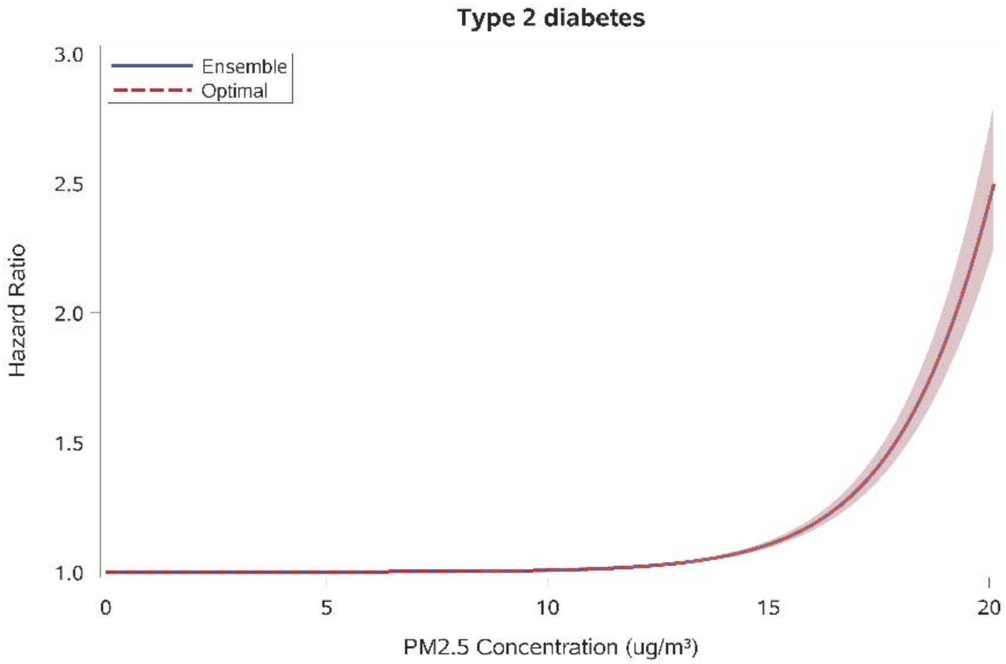

eFigure 4b: Non-linear exposure-response hazard functions for cause-specific mortality (continued). Plots are presented for both the optimal and ensembled model. 95% UI are presented as bands.

© 2019 Bowe B et al. *JAMA Network Open.*

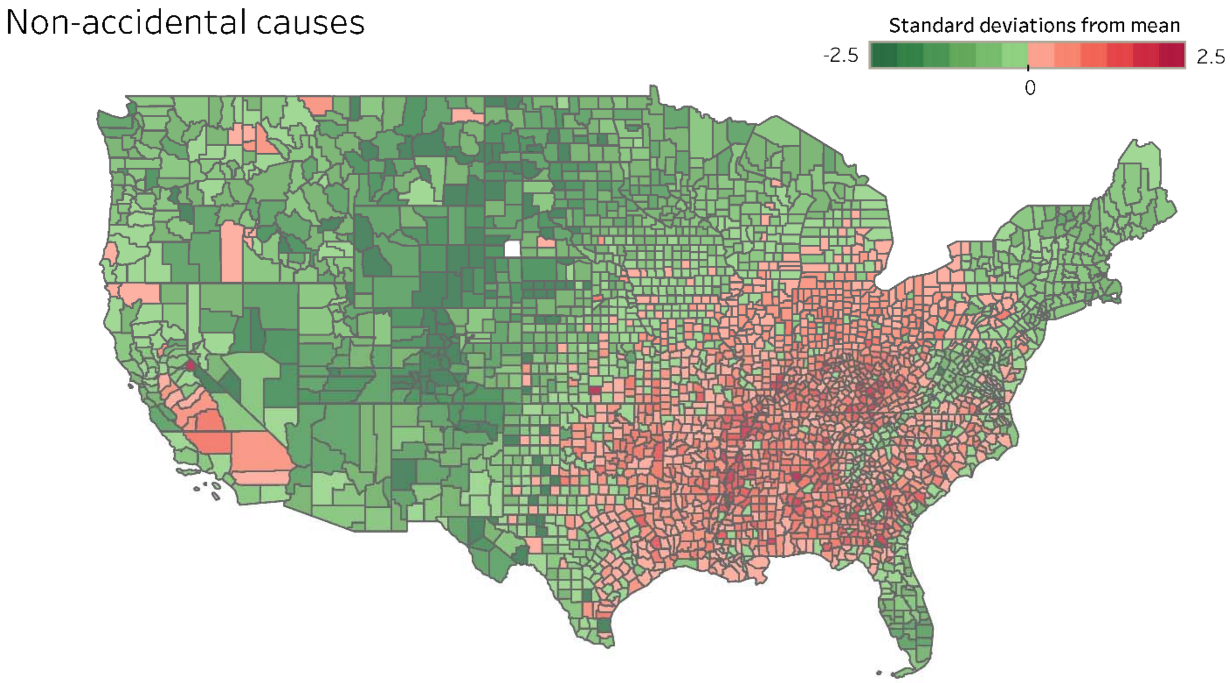

eFigure 5: Map of the age-standardized death rates due to non-accidental causes associated with $PM_{2.5}$ in the contiguous United States by county. Maps are colored by the number of standard deviations from the mean of the states.

© 2019 Bowe B et al. *JAMA Network Open.*

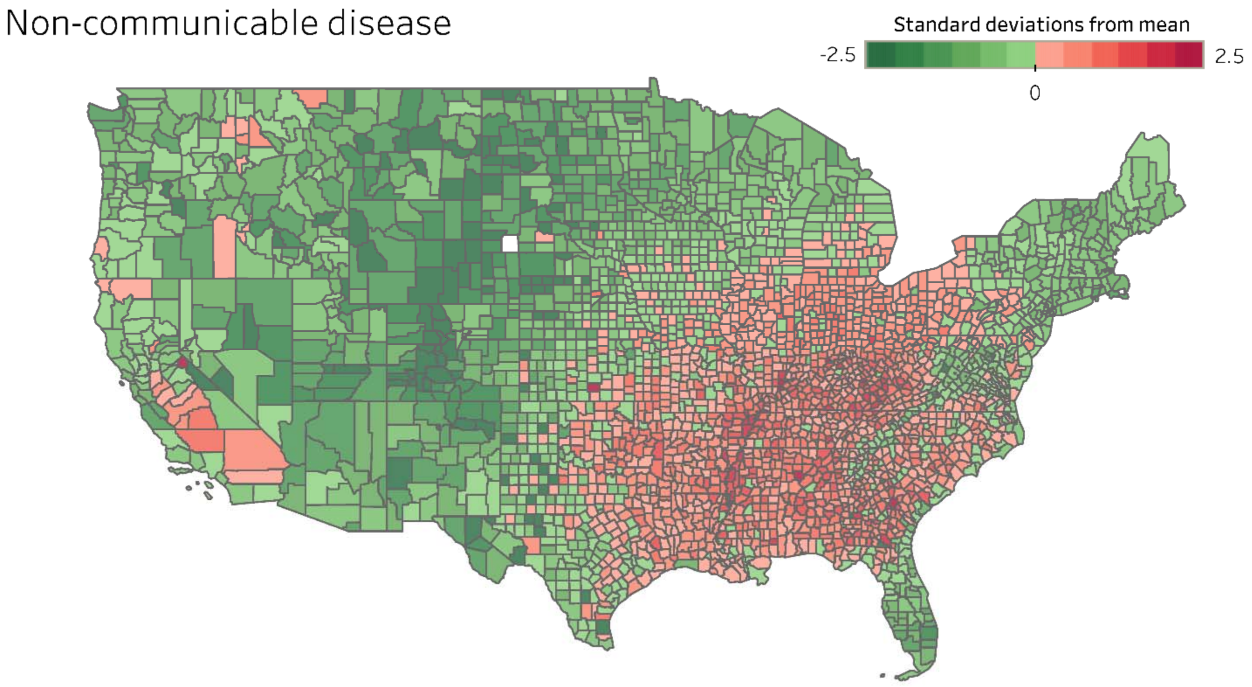

eFigure 6: Map of the age-standardized death rates due to non-communicable diseases associated with $PM_{2.5}$ in the contiguous United States by county. Maps are colored by the number of standard deviations from the mean of the states.

© 2019 Bowe B et al. *JAMA Network Open.*

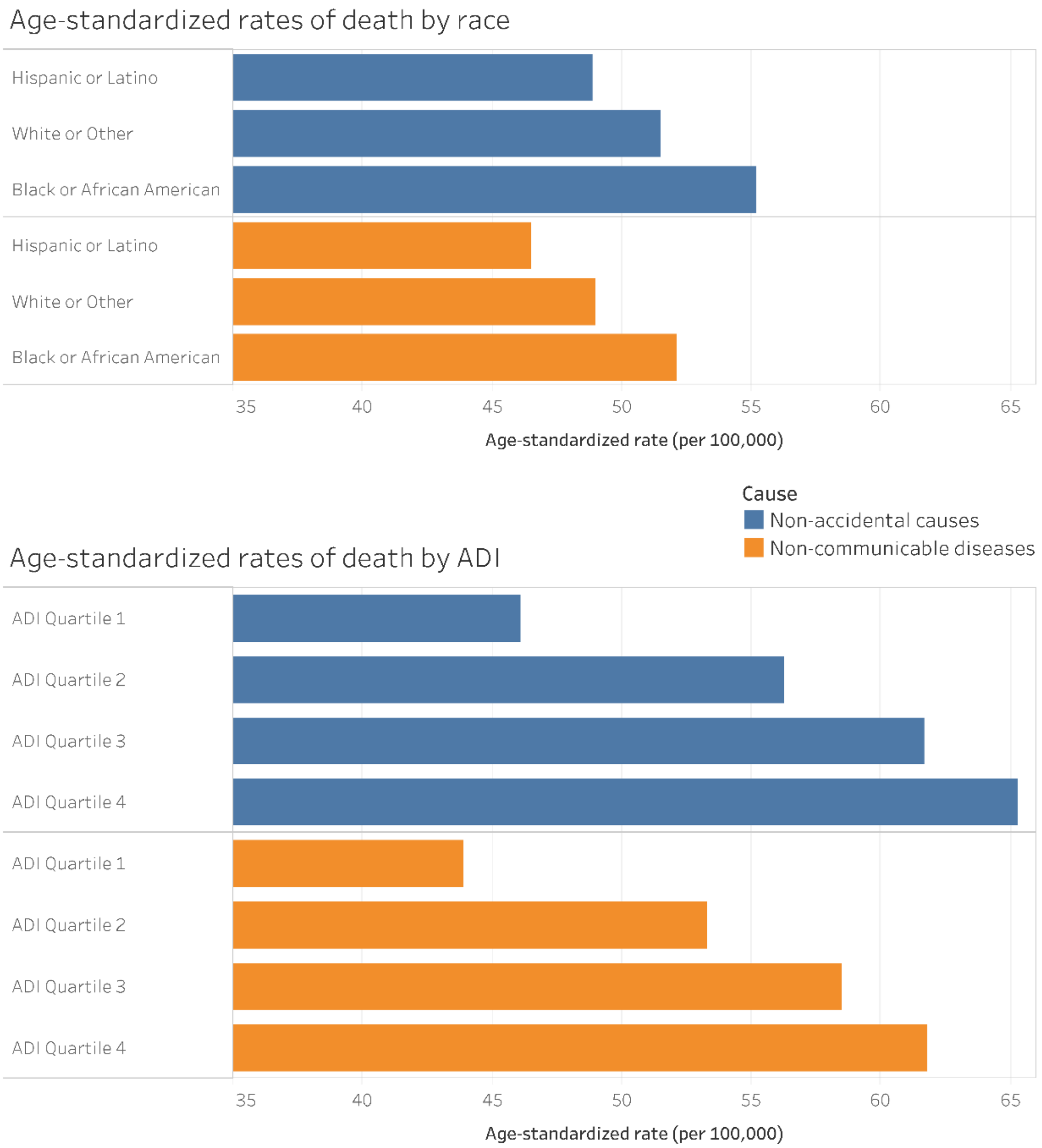

eFigure 7: Age-standardized death rates due to non-accidental causes and non-communicable diseases associated with $PM_{2.5}$ by county Area Deprivation Index and percentage black or African American. Bars are colored by cause of death.

© 2019 Bowe B et al. *JAMA Network Open.*

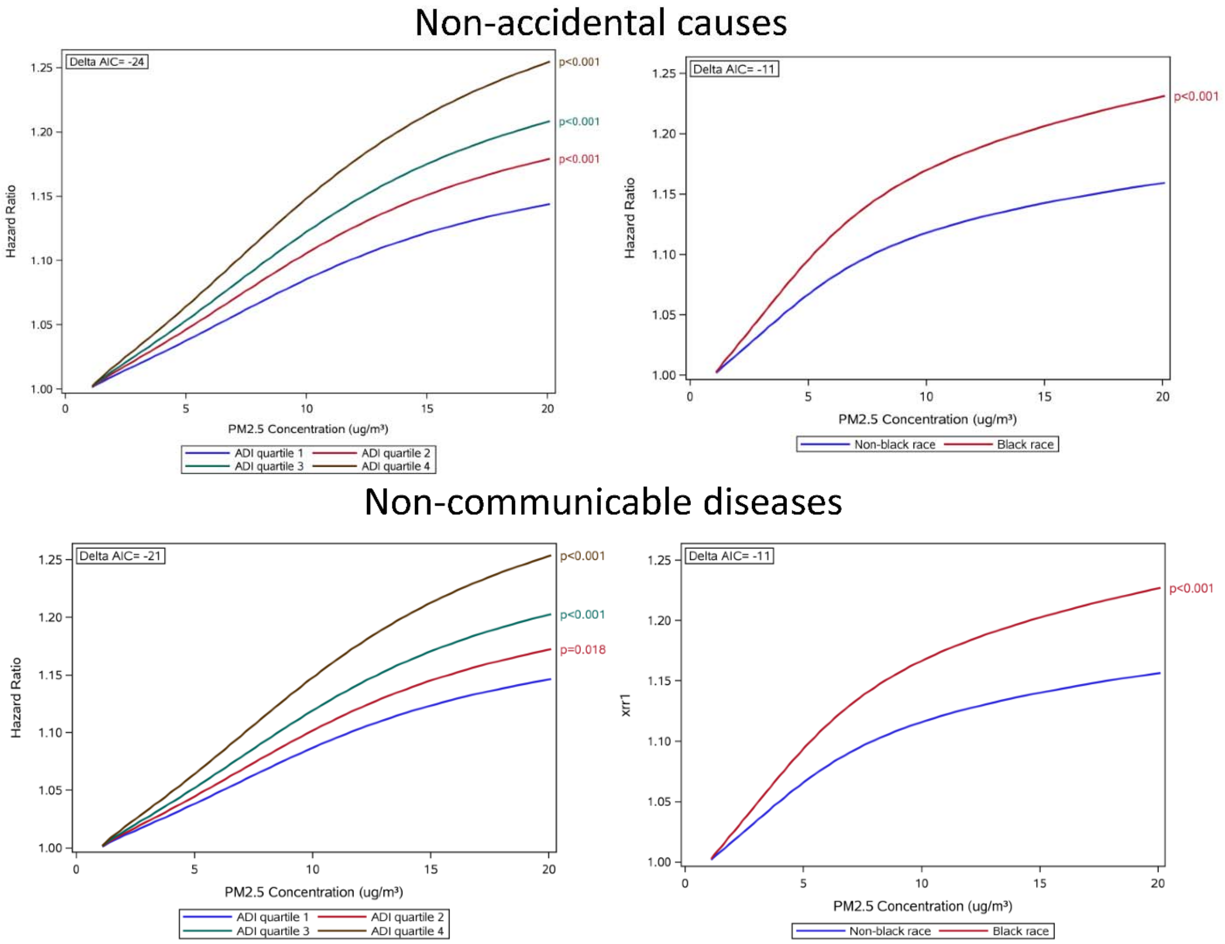

eFigure 8: Effect modification of the association of $PM_{2.5}$ with risk of death due to non-accidental causes and non-communicable diseases by ADI quartile and black race. Results from the optimal model are plotted. Curves are labeled with the p-value of the interaction. The change in AIC versus the non-interaction model is presented.

© 2019 Bowe B et al. *JAMA Network Open.*

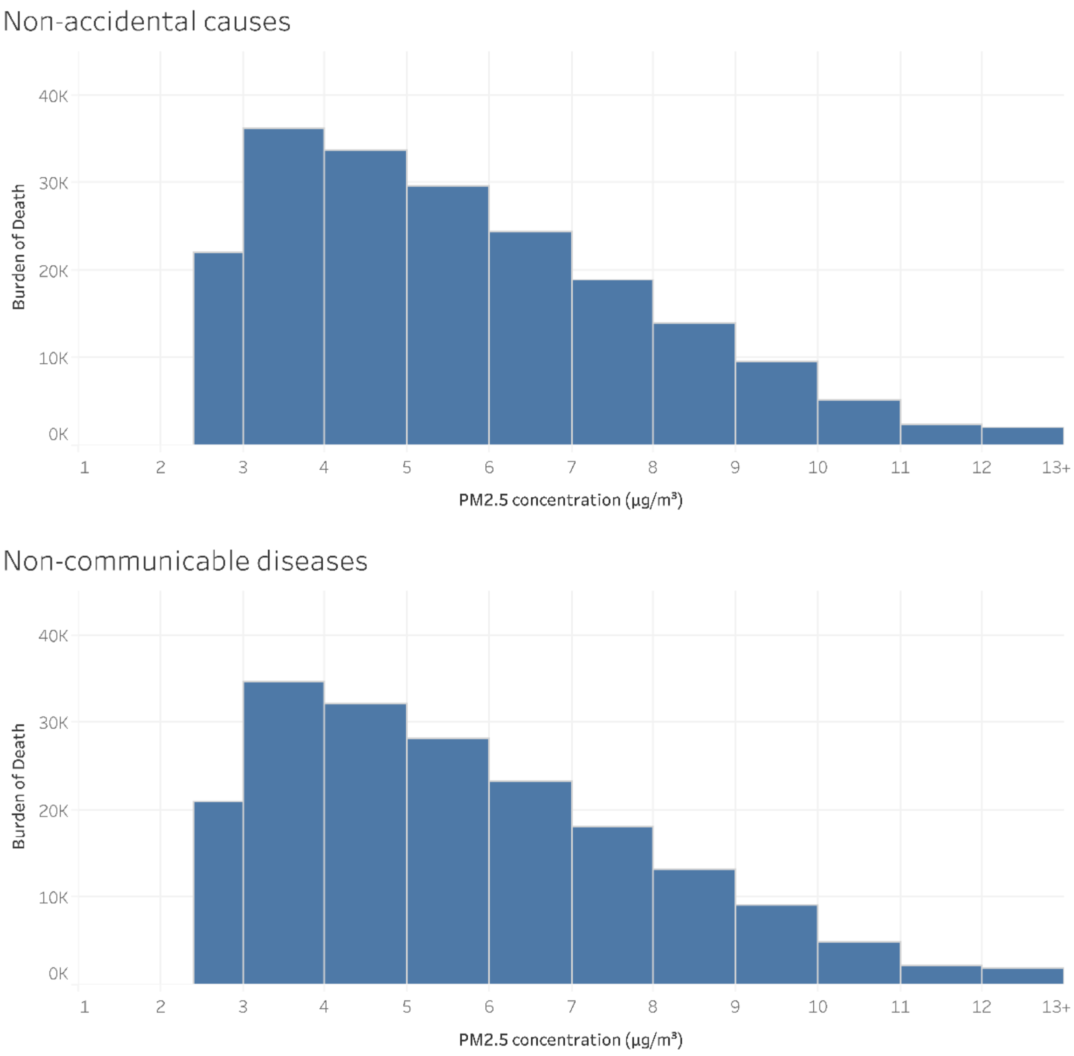

eFigure 9: Burden of death due to non-accidental causes and non-communicable diseases associated with $PM_{2.5}$ in the contiguous US. Bars represent the burden of death associated with $PM_{2.5}$ in the range the bars cover.

© 2019 Bowe B et al. *JAMA Network Open.*

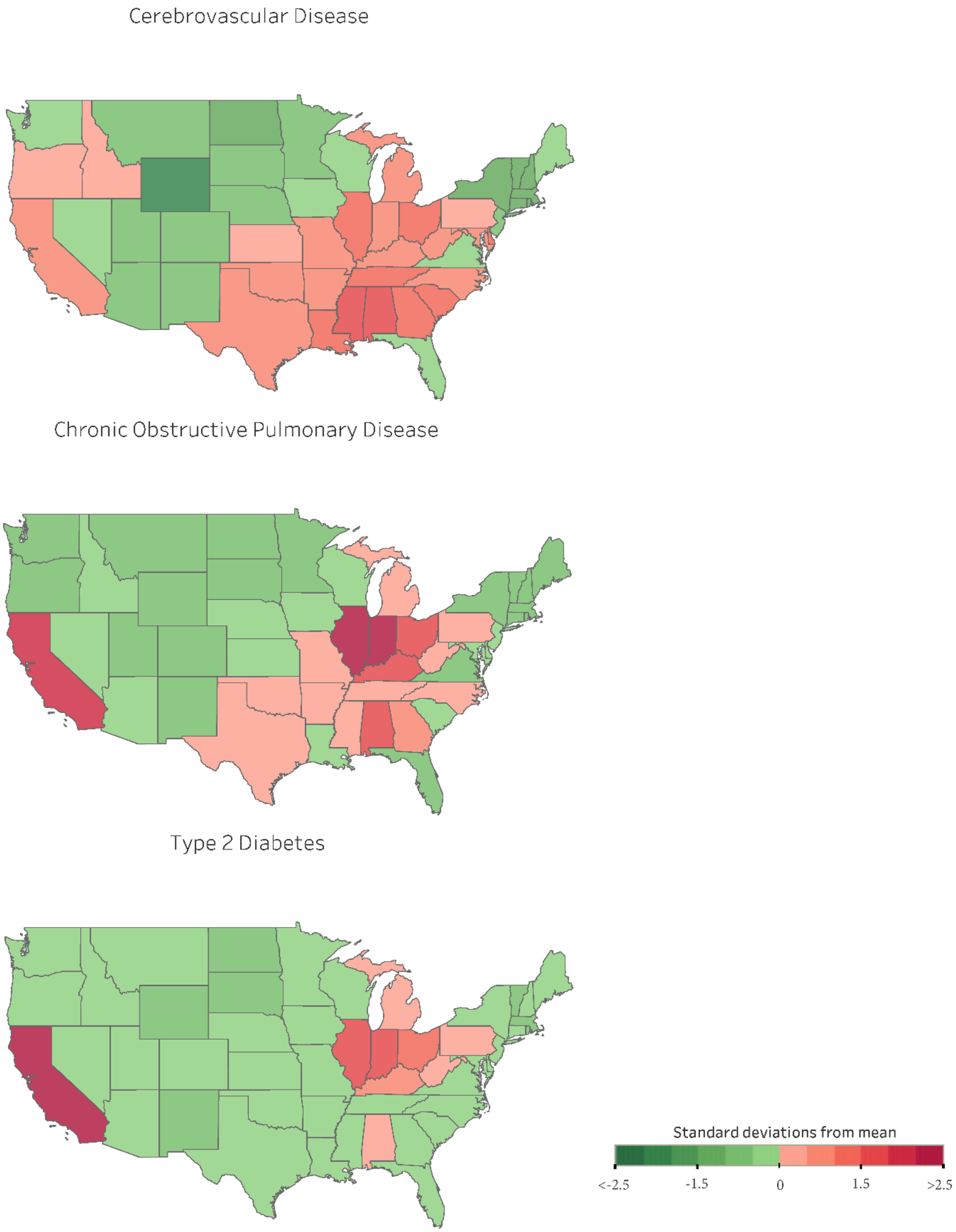

eFigure 10: Maps of the age-standardized death rates due to specific causes associated with $PM_{2.5}$ in the contiguous United States by state (continued). Maps are colored by the number of standard deviations from the mean of the states.

© 2019 Bowe B et al. *JAMA Network Open.*
